# DDX54 drives ALKBH5-mediated demethylation of selected transcripts to suppress interferon antiviral response

**DOI:** 10.1128/jvi.00507-25

**Published:** 2025-08-12

**Authors:** Hao-Yu Sun, Xiu-Ying Gong, Zi-Ling Qu, Li-Li An, Wen-Hao Guo, Hong-Yu Luan, Meng-Yao Wu, Ji-Cheng Yu, Cheng Dan, Yi-Bing Zhang

**Affiliations:** 1 Key Laboratory of Breeding Biotechnology and Sustainable Aquaculture, Institute of Hydrobiology, Chinese Academy of Sciences 53021 , Wuhan, China; 2 University of Chinese Academy of Sciences 74519, Beijing, China; University Medical Center Freiburg, Freiburg, Germany

**Keywords:** DEAD-box helicase 54, ALKBH5, demethylation activity, interferon response, substrate selectivity

## Abstract

DEAD-box (DDX) proteins are currently reported to shape host innate immunity by regulation of N^6^-methyladenosine (m^6^A) modification of transcripts for host factors involved in antiviral signaling. However, which DDX proteins are involved in antiviral response remains incompletely understood. Here, we identified DDX54 as an inhibitor of type I interferon antiviral response by facilitating m^6^A demethylation of selected transcripts. In the presence of VSV infection, DDX54 overexpression downregulated the interferon response and consequently promoted VSV replication, while DDX54 depletion yielded the opposite effects. Knockout of ALKBH5 revealed that DDX54 downregulated the interferon antiviral response through ALKBH5. VSV infection enabled DDX54 relocation from the nucleolus to the nucleoplasm, where DDX54 and ALKBH5 independently bound to the selected m^6^A-modified transcripts forming m^6^A RNA/protein complexes. Although the total enzymatic activity of cellular ALKBH5 was severely impaired in response to VSV infection, DDX54 interaction with ALKBH5 on the selected transcripts promoted the enzymatic activity of ALKBH5, which, in turn, demethylated these selected transcripts, including *mavs* mRNA. Consequently, these selected transcripts were largely retained in the nucleus to limit their translation in the cytoplasm, thereby impairing the interferon antiviral response. ATPase activity was essential for DDX54 to bind the selected transcripts and also to promote ALKBH5 demethylase activity. In the absence of VSV infection, DDX54 failed to recognize cellular m^6^A-modified RNAs and barely promoted the enzymatic activity of ALKBH5. Altogether, we conclude that VSV infection activates a DDX54/m^6^A/ALKBH5 axis to fine tune cellular interferon antiviral response by regulating m^6^A modification of the selected transcripts.

**IMPORTANCE:**

The m^6^A methylation modification on cellular mRNAs affects many cellular processes, including the innate antiviral response. In this study, we reported that VSV infection facilitated RNA helicase DDX54 to relocate from the nucleolus to the nucleoplasm, where DDX54, together with the m^6^A eraser ALKBH5, bound to a common subset of m^6^A-modified transcripts for host factors involved in antiviral signaling. Such binding promoted the enzymatic activity of ALKBH5 to demethylate the m^6^A modification on these transcripts, therefore limiting their protein translation and consequently impairing interferon antiviral response. Our results reveal an inhibitory function of DDX54 on host innate antiviral response.

## INTRODUCTION

Interferon (IFN)-mediated innate immune response constitutes the primary line of defense against viruses and is initiated by host pattern recognition receptors (PRRs) recognizing virus infection (1). The well-characterized PRRs include retinoic acid-inducible gene-I (RIG-I)-like receptors (RLRs) that are composed of RIG-I, melanoma differentiation-associated antigen 5 (MDA-5), and laboratory of genetics and physiology 2 (LGP2), and they recognize viral dsRNA in the cytoplasm of both immune and nonimmune cells. Such recognizing signaling is quickly transmitted to mitochondrial antiviral-signaling protein (MAVS), a key adaptor involved in RNA virus infection. MAVS subsequently recruits a key protein kinase TANK-binding kinase 1 (TBK1), resulting in phosphorylation and activation of interferon regulatory factor 3 (IRF3) and IRF7, which, in turn, translocate to the nucleus and trigger IFN expression. IFNs subsequently upregulate the expression of hundreds of cellular IFN-stimulated genes (ISGs) to clear virus invading (1). Despite the necessity, IFN production during virus infection is tightly regulated to prevent harmful immunopathology caused by excessive IFN expression and also avoid insufficient virus clearance caused by less expression (2).

Emerging evidence has shown that the posttranscriptional modification plays essential roles in regulating the innate antiviral response (3–5). Methylation at the N^6^ position of adenosine (m^6^A), the most prevalent internal modification in eukaryotic mRNAs, is a dynamic and reversible process, which is cooperatively controlled by m^6^A machinery that is composed of m^6^A writers, readers, and erasers (6). Generally, m^6^A is co-transcriptionally deposited on mRNAs by usually m^6^A methyltransferase-like 3 (METTL3)/METTL14 heterodimer and additional adaptor such as WT1-associated protein (WTAP) as m^6^A writers (7) and is dynamically removed from m^6^A-modified mRNAs by two currently known demethylases (erasers), obesity-associated protein (FTO), and AlkB homolog 5 (Alkbh5) (8). m^6^A readers are several YTH domain-containing RNA-binding proteins that have a potential to specifically recognize m^6^A-modified RNAs (YTHDC1, YHTDC2, YTHDF1, YTHDF2, and YTHDF3), and such recognition directly affects many aspects of cellular gene expression, including mRNA decay, mRNA splicing, mRNA nuclear export, and translation, which is often dependent on different readers and their cellular localizations under a given condition (4–6).

In virus-infected cells, cellular RNA methylomes are heavily influenced (5). Individual knockout of key components of the cellular m^6^A machinery, e.g., ALKBH5 (9), METTL3 (10–12), METTL4 (13, 14), and YTHDF2 (10), often improves host antiviral immunity. These results indicate that cellular RNA m^6^A modification might serve as a negative regulator of host antiviral response. Consistently, type I IFN signaling is often targeted for virus-directed m^6^A methylation. For example, MAVS mRNA undergoes METTL14-mediated m^6^A modification upon RNA virus infection, thus facilitating its mRNA decay (14). WTAP-mediated m^6^A modification leads to translation suppression of IRF3 mRNA and instability of IFNAR1 mRNA (15). IFNB mRNA is also m^6^A-modified and is stabilized by depletion of METTL3 or YTHDF2 (10). How these transcripts are selectively regulated for m^6^A modification remains incompletely understood.

It is hypothesized that some RNA helicase might drive m^6^A modification of selective genes to fine tune the antiviral innate immunity (9, 16). In response to vesicular stomatitis virus (VSV) infection, nuclear DEAD-box helicase 46 (DDX46) recruits ALKBH5 to demethylate some m^6^A-modified transcripts for host factors involved in antiviral signaling (9). DDX5 promotes m^6^A methylation and nuclear export of transcripts *p65* and *ikkg*, but both transcripts undergo a YTHDF2-dependent mRNA decay (16). All DDX helicases contain a conserved core RNA helicase domain with ATPase activity and RNA-binding activity, which is thought to be essential for transcriptional regulation (17, 18).

DDX54 is characterized as a nucleolus RNA helicase and is essential for formation of the ribosome active site by remodeling rRNA structure (19); in response to ionizing radiation, DDX54 undergoes a nucleolus-to-nucleoplasm translocation to regulate transcriptome dynamics (20). Here, we reported that DDX54 acted as a negative regulator of cellular IFN response. In response to VSV infection, DDX54 translocated from the nucleolus to the nucleoplasm, where DDX54 interacted with ALKBH5 on a common subset of m^6^A-modified transcripts involved in RLR signaling. Such interaction significantly promotes the demethylase activity of ALKBH5 to erase m^6^A methylation of these selected antiviral molecules, dampens their nuclear exporting and protein translation in cytoplasm, and, therefore, suppresses cellular IFN antiviral response. Our findings provide an insight into how DDX54 guides ALKBH5 to demethylate the selected transcripts and highlight a regulatory role of the DDX54/m^6^A/ALKBH5 axis in balancing type I IFN response.

## MATERIALS AND METHODS

### Cells, virus, and antibodies

HEK293T and H1299 cell were originally obtained from American Type Culture Collection (ATCC) and were cultured in DMEM with 10% fetal bovine serum (FBS) at 37℃ in a humidified incubator containing 5% CO_2_. VSV and VSV-GFP were proliferated in Vero cells as described previously (21). DDX54 antibody was generated by DIA-AN Technology (Wuhan, China). The antibodies specific to ALKBH5 (A11684), m^6^A (A19841), MAVS (A5764), TBK1 (A3458), IRF7 (A0159), TRAF3 (A3094), and TRAF6 (A16991) were obtained from Abclonal Inc. company (Wuhan, China), and the antibodies specific to IRF3 (#11904), HA (#3724), and Flag (#A70569) were obtained from Cell Signaling Technology (Danvers, MA, USA).

### Plasmids

The ORFs of *ddx54* (NM_024072.4)*, alkbh5* (NM_017758.3)*, mettl3* (NM_019852.5)*, mettl14* (NM_020961.4)*, mavs* (NM_020746.4), and *tbk1 *(NM_013254.3) were inserted into the *EcoR* V site of pcDNA3.1(+) vector for untagged expression plasmids. Flag- and HA-tagged expression plasmids were made by cloning Flag- or HA-fused ORFs into the *EcoR* V site of pcDNA3.1(+). If needed, the corresponding 3′UTR sequences were included in the untagged MAVS and TBK1 plasmids as mentioned above. DDX54 mutants (D250A, TG280G, G401A) were made by point-mutation of the corresponding amino acids. DDX54-GFP was made by inserting the corresponding ORF into the *EcoR*I site of pEGFP-N3.

### Cell transfection, VSV-GFP replication assays, and luciferase assays

Cell transfection was performed as previous description (22, 23). Briefly, cells were transfected with the indicated expression plasmids, which were diluted into Opti-MEM (Thermo Fisher Scientific) and mixed with polyethylenimine (PEI, molecular mass 25,000 Da, Sigma-Aldrich; 1 µg/mL of storage concentration) at a ratio of 1:5 plasmid/PEI (μg/μL).

For VSV-GFP replication assays, cells seeded in 6-well plates overnight were transfected with DDX54 or ALKBH5 expression plasmids or the derived mutant plasmids. Twenty-four hours later, cells were infected with VSV-GFP. Another 12 h later, green signaling was captured by fluorescence microscope, and the percentage of VSV-GFP-infected cells in total cells was measured by ImageJ software (Version 1.53).

Luciferase activity assays were performed as previously reported (24, 25). Typically, cells seeded in 48-well plates overnight were transfected with IFNβ promoter-driven luciferase plasmid (IFNβpro-luc), the indicated expression plasmid, and Renilla luciferase plasmid pRL-TK at a ratio of 1000:1000:1 for 24 h. If needed, cells were infected with VSV or transfected with poly(I:C) (I3036, SIGMA). Another 6 h later, cells were harvested to detect the luciferase activity on Junior LB9509 luminometer (Berthold, Pforzheim, Germany) by Dual-Luciferase Reporter Assay System (Promega, USA).

### CRISPR-Cas9 knockout cell lines

DDX54 and ALKBH5 knockout HEK293T cell line was constructed by the clustered regularly interspaced short palindromic repeats (CRISPR)/Cas9 technology. Single-guide RNA (sgRNA) targeting sequences (for DDX54: 5′-GGCGAGTTTGAGATCCAGGC-3′, 5′-GTCTGGAGGCTTCCAGTCCA-3′, 5′-AGGTGCCAACACCCATCCAG-3′, and 5′-ATCTTGGATGGCAAGGACGT-3′; for ALKBH5: 5′-TCCCGGGACAACTATAAGGC-3′, 5′-GGACACAGGGTAAGGTTCGG-3′, and 5′-CGTGGACGAGATCCCCGAGT-3′) were designed using the MIT online tool (http://crispr.mit.edu/) and then cloned into Cas9-expressing lentiviral transfer vector (lentiCRISPRv2) with the MultiF seamless assembly kit (Abclonal, RK21020). LentiCRISPRv2 plasmids were transfected with viral packaging plasmids (YEASEN, China) into HEK293T cells. Two days later, cells were harvested, and viral supernatant was used to infect HEK293T cells in the presence of Polybrene (8 mg/mL) for 48 h, followed by selectively culturing in puromycin (2 mg/mL)-containing cell medium for 2 weeks. Single knockout cell was picked by Flow cytometry (Beckman, Molfo-XDP, USA) and serially culturing in 96-wells plates to generate a stable cell line. The knockout efficiency was determined by genomic DNA sequencing and western blots. During cell maintaining, we found that the KO cells are nearly normal, except for having a slightly slow growth rate compared to WT cells.

### RNA fractionation, RNA extraction, and RT-qPCR

Nuclear and cytoplasmic RNA separation was performed according to a previous report (9). Briefly, cells were washed twice with ice-cold PBS buffer and were lysed for 30 min with ice-cold lysis buffer (10 mM Tris-HCl, 140 mM NaCl, 1.5 mM MgCl_2_, 10 mM EDTA, 0.5% NP-40, and 40 U/µlLRNasin, pH7.4). Cell lysate was separated by centrifugation into a supernatant containing cytoplasmic RNA and a precipitate containing nuclear RNA, the latter of which was rinsed with ice-cold lysis buffer twice followed by further centrifugation to discard the supernatants. Cytoplasmic and nuclear RNAs were then extracted by TRIZOL reagent (TIANMO BIOTECH, China). Single-stranded cDNA was synthesized using the First-Strand Synthesis System (Monad, China). GAPDH and RNU6 were used as the internal indicators of cytoplasmic and nuclear RNA, respectively. RT-qPCR was performed using Universal Blue qPCR SYBR Green Master MIX (TEASON, China) on a Real-Time System (Bio-Rad, CA, USA). The primer sequences used in this study are listed in Table S1.

### m^6^A RNA content measurement

Total RNA was extracted with the TRIzol reagent followed by quantification, and the m^6^A content in total RNA was measured using the Colorimetric EpiQuik m^6^A RNA Methylation Quantification Kit (P-9005, Epigentek, USA) according to the manufacturer’s instructions. Briefly, equal amounts of total RNAs were bound to strip wells in a 96-well plate by incubation with RNA-binding solution at 37°C for 90 min. After RNA binding, the m^6^A RNA in strip wells was captured by incubation with capture antibody solution for 60 min, followed by incubation with detection antibody buffer and enhancer solution for 30 min. The detected signal was then enhanced by incubation with color developing solution for color development and finally quantified colorimetrically by reading the absorbance in a microplate spectrophotometer at 450 nm within 10 min.

### Co-immunoprecipitation and western blotting

Co-IP assays and western blotting were performed as described previously (26). Cells under different treatments were lysed with NP-40 lysis buffer (Beyotime, China). Cell lysates were incubated with anti-Tag magnetic beads at 4°C overnight, and under some conditions, DDX54 or ALKBH5 Ab-conjugated protein A/G magnetic beads were used instead of anti-Tag magnetic beads. The beads were washed with Co-IP wash buffer (50 mM Tris-HCl, pH 7.5, 150 mM NaCl, 1 mM DTT, 1% NP-40), resolved in SDS loading buffer (Biosharp, China), followed by western blotting with the indicated antibodies.

### RNA-binding protein immunoprecipitation combined with qPCR

RNA-binding protein immunoprecipitation (IP) assays were performed as described previously (27). Briefly, HEK293T cells seeded in 10 cm^2^ plates overnight were transfected with the indicated plasmids for 24 h, and if needed, cells were further infected with VSV infection for 6 h. After treatments, cells were lysed with ice-cold RIP lysis buffer (50 mM Tris-HCl [pH 7.5], 150 mM NaCl, 0.5% Triton X-100, 10% glycerol, 1 mM EDTA and 40 U/µL RNasin) at 4°C for 30 min, followed by centrifugation at 4°C for 10 min. Cell supernatants were incubated at 4℃ for 4 h with the indicated IP Abs that were pre-conjugated to Protein A/G magnetic Beads (Mabus, China). The immunoprecipitated RNA-protein complexes were subjected to extract total RNAs by the TRIZOL reagent (TIANMO BIOTECH, China), followed by RT-qPCR. The relative mRNA enrichment was normalized to the corresponding input.

### m^6^A RNA immunoprecipitation combined with qPCR or western blotting

m^6^A RNA IP assays were performed as the above-mentioned RNA-binding protein IP assays, just with anti-m^6^A RNA Ab instead of a protein Ab as the IP Ab. Before IP experiments, anti-m^6^A Ab or IgG was conjugated with Protein A/G magnetic Beads (Mabus, China) magnetic beads at 4°C for 2 h. After cell supernatants were incubated with IP Ab-conjugated beads at 4°C for 6 h, the immunoprecipitated RNA-protein complexes were subjected to RNA extraction for RT-qPCR analysis of the indicated mRNA expression, or to SDS-PAGE electrophoresis for western blotting analyses of DDX54 or ALKBH5 protein levels in these complexes.

### m^6^A demethylation activity of ALKBH5

m^6^A demethylation activity of cellular ALKBH5 was performed with the Epigenase m^6^A Demethylase Activity/Inhibition Assay Kit (P-9013, EpiGentek, USA) according to the manufacturer’s instructions. Briefly, HEK293T cells seeded in 10 cm^2^ plate overnight were transfected with ALKBH5, together with DDX54, D250A, or empty vector (5 µg each) as control. Twenty-four hours later, cells were infected with or without VSV (MOI = 1). Another 6 h later, cells were scraped carefully into 0.9% physiological saline by a cell scraper, followed by purification of cellular ALKBH5 protein with anti-ALKBH5 Ab. Equal amounts of ALKBH5 protein samples were incubated with the unique m^6^A substrate in 50 µL of reaction mixture containing (NH_4_)_2_Fe(SO_4_)_2_·6H_2_O (150 µM), KCl (100 mM), MgCl_2_ (2 mM), α-KG (300 µM), L-ascorbic acid (2 mM), and HEPES buffer (50 mM, pH 7.0). Two hours later, the signal was colorimetrically quantified through microplate reader at 450 nm with an optional reference wavelength of 655 nm.

### ATPase activity of DDX54 and D250A

To detect the possible effects of ATPase activity of DDX54, the ATPase activity of DDX54 and D250A was measured with ATPase Activity Assay Kit (E-BC-K831-M, Elabscience, China) Briefly, HEK293T cells seeded in 10 cm^2^ plate overnight were transfected with DDX54, D250A, or empty vector (5 µg each) as control. Thirty-six hours later, cells were scraped for nuclear protein extraction by the Nuclear and Cytoplasmic Protein Extraction Kit (20126ES60, YEASON, China). According to the manufacturer’s instructions of ATPase Activity Assay Kit, the nuclear protein was incubated with ATP substrate and reaction buffer at 37°C for 30 min, generating inorganic phosphate that was hydrolyzed from ATP. The signal was colorimetrically quantified based on the amounts of inorganic phosphate through microplate reader at 640 nm.

### Immunofluorescence analysis and imaging

Cells were seeded on coverslip in 6-well plates with cover slip for 12 h, followed by plasmids transfection or VSV infection. Cells were fixed with 4% (vol/vol) paraformaldehyde (PFA) in PBS at 4°C overnight. After washing three times, cells were incubated with 0.2% triton X-100 for 15 min and stained with DAPI (Beyotime) for 15 min. After PBS washing, the samples were immersed with the indicated Ab, followed by incubation with fluorescent-dye-conjugated secondary Ab. The images were obtained by Axio Imager M2 fluorescence motorized microscope (Carl Zeiss) and analysis with software, ZEN 2.3 (Blue edition).

### Statistical analysis

All experiments were replicated at least three times. A Student *t* test was applied for statistical analysis of the data. “ns” indicates no significant difference (*P* > 0.05). *, **, and *** indicate statistically significant differences with values of *P* < 0.05, *P* < 0.01, and *P* < 0.001, respectively.

## RESULTS

### Overexpression of DDX54 promotes VSV replication and impairs IFN response

**Fig 1:**
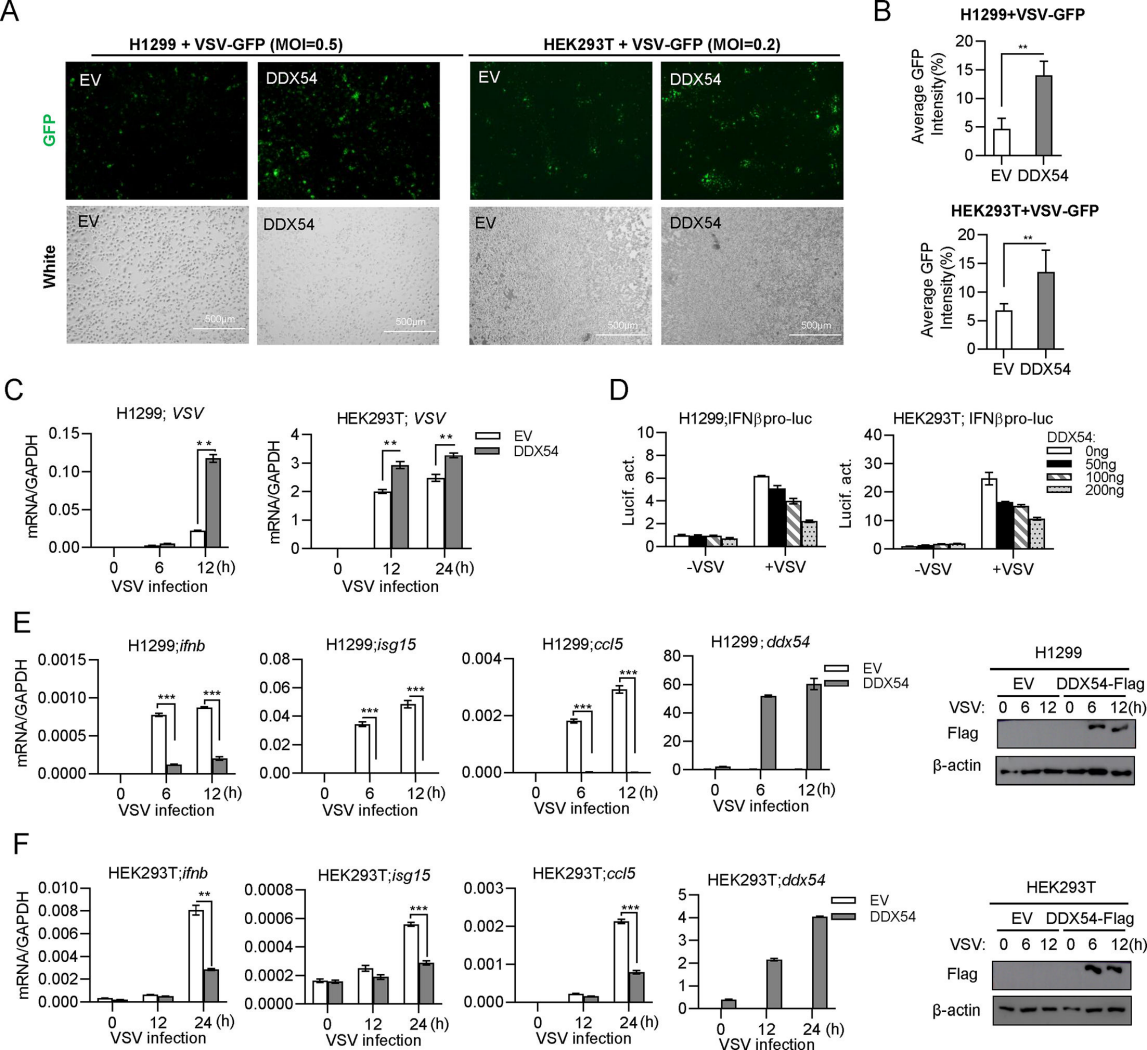
Overexpression of DDX54 promotes virus replication and impairs IFN response. (**A–C**) Overexpression of DDX54 promoted VSV replication in both H1299 and HEK293T cells. H1299 or HEK293T cells seeded in 6-well plates were transfected for 24 h with empty vector (EV) and DDX54 (100 ng each), followed by infection with VSV-GFP (**A and B**) or with VSV (**C**). Another 12 h later, cells were visualized by fluorescence microscope (**A**) or subjected to evaluate virus replication by software ImageJ to count the ratio of GFP positive/negative cells (**B**). Or cells were collected for RT-PCR analyses of VSV genome RNAs at the indicated times (**C**). (**D**) Overexpression of DDX54 inhibited IFNβ protomer activation by VSV infection. H1299 or HEK293T cells seeded in 48-well plates overnight were transfected for 24 h with IFNβpro-luc (100 ng), pRL-TK (1 ng), and DDX54 at increasing doses (0, 10, 50, 100 ng), followed by infection with VSV (MOI = 1). At 6 h post infection, cells were harvested for luciferase assays. (**E and F**) Overexpression of DDX54 inhibited mRNA expression of IFN and ISGs by VSV infection. H1299 cells (**E**) and HEK293T cells (**F**) seeded in 12-well plates were transfected with EV or DDX54 (500 ng) for 24 h, followed by infection with VSV (MOI = 0.5 and 1, respectively). At the indicated time points, cells were collected for RT-qPCR analysis of *ddx54, ifnb, isg15,* and *ccl5*. Data were normalized to GAPDH. The overexpression of DDX54 protein was detected by western blotting analysis. Error bars represent SDs obtained by measuring each sample in triplicate. **P < 0.01, ***P < 0.001, Student *t* test. The results are representatives of at least three independent repeats.

To determine the role of DDX54 in the innate antiviral response, H1299 and HEK293T cells were individually transfected with DDX54, followed by VSV-GFP challenge. Virus production was illustrated by counting the ratio of GFP positive/negative cells. Compared with control cells transfected with empty vector (EV), DDX54-transfected cells exhibited an enhanced green fluorescence (Fig. 1A), as supported by quantitation of GFP intensities (Fig. 1B). RT-PCR analyses revealed a higher level of VSV genome RNA in DDX54-transfected cells than EV-transfected cells (Fig. 1C). Therefore, DDX54 overexpression can promote VSV replication.

Subsequent assays were performed to determine whether DDX54 was able to regulate the IFN response. Luciferase assays showed a robust activation of IFNβ promoter-driven luciferase reporter plasmid (IFNβpro-luc) by VSV infection in H1299 or HEK293T cells, but this activation was inhibited by overexpression of DDX54 in a dose-dependent manner (Fig. 1D). Consistently, VSV infection induced mRNA expression of cellular *ifnb, isg15,* and *ccl5* genes; however, this induction was significantly suppressed by overexpression of DDX54 in H1299 cells (Fig. 1E) and HEK293T cells (Fig. 1F). Therefore, DDX54 overexpression impairs the IFN response induced by VSV infection.

### Knockout of DDX54 inhibits VSV replication and enhances IFN response

**Fig 2:**
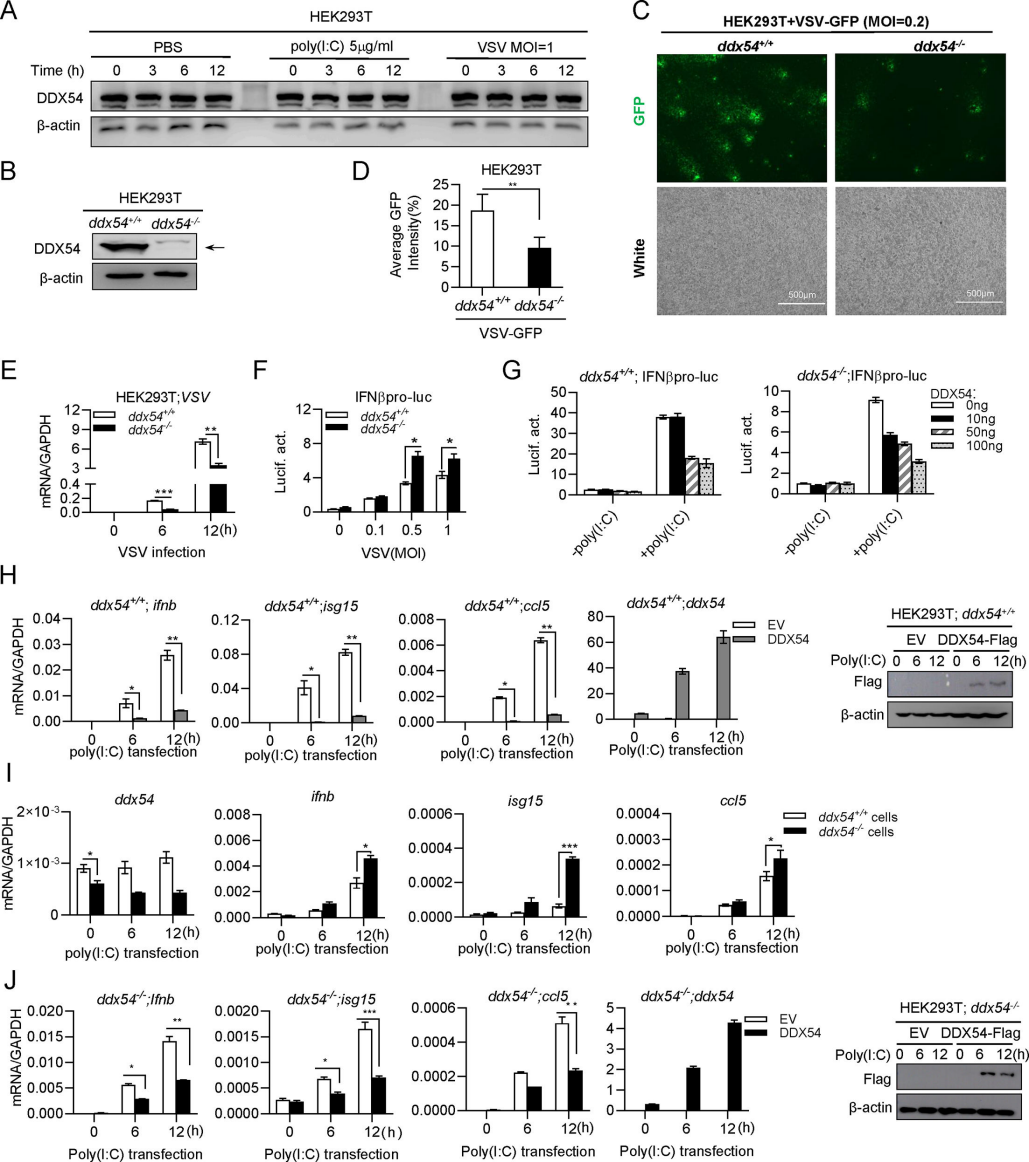
Knockout of DDX54 inhibits virus replication and enhances IFN response. (**A and B**) Western blotting analyses of endogenous DDX54 protein in HEK293T cells following poly(I:C) transfection or VSV infection (**A**), or in wildtype (*ddx54^+/+^*) and *ddx54^−/^*^−^ HEK293T cells (**B**). (**C–E**) Knockout of DDX54 inhibited VSV replication. *ddx54^+/+^* and *ddx54^−/^*^−^ HEK293T cells seeded in 6-well plate overnight were infected by VSV-GFP (C and D, MOI = 0.2) or VSV (E, MOI = 1). Twelve hours later, cells were visualized by fluorescence microscope (**C**), and virus replication was evaluated by calculating the ratio of GFP positive/negative cells with software ImageJ (**D**). Or cells were collected for RT-PCR analyses of VSV genome RNA at the indicated times (**E**). (**F and G**) Knockout of DDX54 promoted IFNβ protomer activation by VSV (**F**) or poly(I:C) (**G**). *ddx54^+/+^* or *ddx54^−/^*^−^ cells seeded in 48-well plates overnight were transfected with IFNβpro-luc (100 ng) and pRL-TK (1 ng) (**F**), or together with DDX54 at increasing doses (0, 10, 50, 100 ng) (**G**).Twenty-four hours later, cells were infected with VSV (F, MOI = 1) or transfected with for poly(I:C) (5 µg/mL) (**G**). Another 12 h later, cells were harvested for luciferase assays. (**H–J**) DDX54 inhibited mRNA expression of IFN and ISGs by poly(I:C). *ddx54^+/+^* cells (**H**), or both *ddx54^+/+^* and *ddx54^−/^*^−^ cells (**I**), or *ddx54^−/^*^−^ cells (**J**), seeded in 12-well plates, were transfected with EV or DDX54 (500 ng) for 24 h, followed by transfection with poly(I:C) (5 µg/mL). At the indicated time points, cells were collected for RT-qPCR analysis of *ddx54, ifnb, isg15,* and *ccl5*. Data were normalized to GAPDH. The overexpression of DDX54 protein was detected by western blotting analysis. Error bars represent SDs obtained by measuring each sample in triplicate. *P < 0.05, **P < 0.01, ***P < 0.001, Student *t* test. The results are representatives of at least three independent repeats.

We wondered whether knockout of DDX54 could counteract its inhibitory effect on virus replication and cellular IFN response. DDX54 protein was constitutively expressed in HEK293T cells regardless of poly(I:C) transfection or VSV infection (Fig. 2A). We used the CRISPR/Cas9 editing technology to knockout DDX54 gene in HEK293T cells (Fig. 2B), which was verified by genomic DNA sequencing (Fig. S1A). DDX54-deficient cells (*ddx54^−/^*^−^) were more sensitive to VSV-GFP infection than wild-type cells (*ddx54^+/+^*) (Fig. 2C), showing a high level of GFP intensity (Fig. 2D) as well as a high level of VSV genome RNAs in *ddx54^−/^*^−^ cells (Fig. 2E). In addition, VSV infection activated IFNβ promoter more significantly in *ddx54^−/^*^−^ cells than in *ddx54^+/+^* cells (Fig. 2F).

We repeated these assays by poly(I:C) transfection instead of VSV infection. It showed that poly(I:C)-triggered activation of IFNβ promoter was dose-dependently inhibited by overexpression of DDX54 in either *ddx54^+/+^* cells or *ddx54^−/^*^−^ cells (Fig. 2G). Similarly, poly(I:C)-triggered mRNA expression of cellular *ifnb, isg15,* and *ccl5* was time-dependently attenuated by overexpression of DDX54 in *ddx54^+/+^* cells (Fig. 2H). Compared with *ddx54^+/+^* cells, *ddx54^−/^*^−^ cells had a high mRNA expression of these genes by poly(I:C) (Fig. 2I). Therefore, DDX54 promotes VSV replication likely through impairing cellular IFN response.

### DDX54 relocates from the nucleolus to the nucleoplasm upon VSV infection

**Fig 3:**
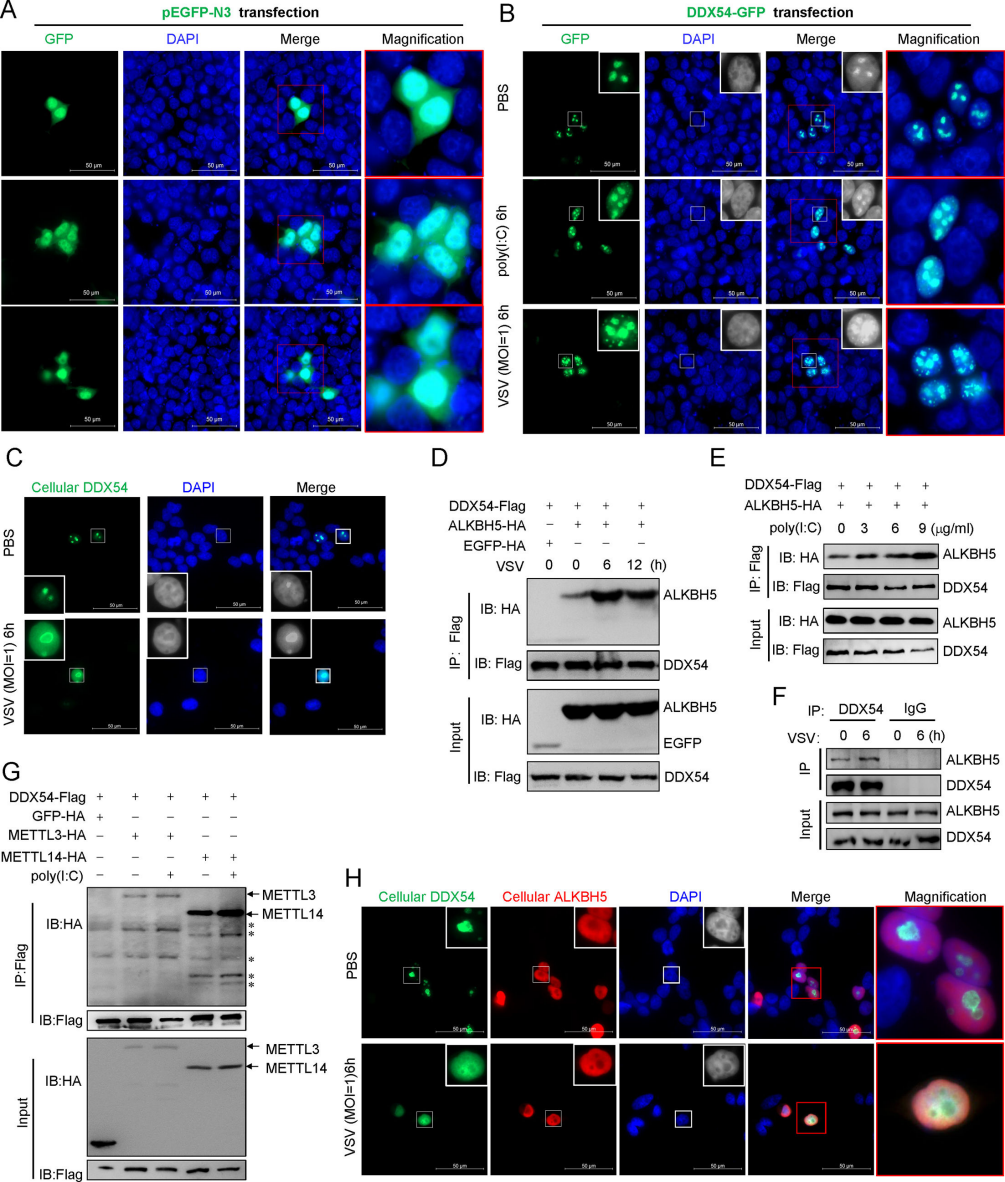
DDX54 interacts with ALKBH5 in the nucleoplasm after viral infection. (**A–C**) Relocation of DDX54 protein from the nucleolus to the nucleoplasm upon poly(I:C) or VSV. HEK293T cells seeded in 6-well plates with cover slip overnight were transfected for 24 h with pEGFP-N3 (**A**) or DDX54-GFP (**B**), followed by transfection with poly(I:C) (5 µg/mL) or infection with VSV (MOI = 1). Another 6 h later, cells were visualized by fluorescence microscope (**A and B**). Or HEK293T cells were directly infected with VSV for 6 h, followed by immunofluorescence assays with DDX54 antibody (**C**). (**D–F**) Co-IP analyses of the interaction of DDX54 with ALKBH5. HEK293T cells seeded in 10 cm^2^ plates overnight were transfected for 24 h with DDX54-Flag and ALKBH5-Flag (5 µg each), followed by VSV infection (MOI = 1) (**D**) or poly(I:C) transfection (**E**). Or cells were directly infected with VSV (**F**). Another 6 h later, cells were collected for Co-IP assays with tag-specific antibody (**D and E**) or with DDX54 antibody (**F**). (**G**) Co-IP analyses of the interaction of DDX54 with METTL3 or METTL14. HEK293T cells seeded in 10 cm^2^ plates overnight were transfected for 24 h with the indicated plasmids (5 µg each), followed by poly(I:C) transfection (5 µg/mL). At 6 h post transfection, cells were collected for Co-IP assays with tag-specific antibody. * indicates “non-specific binding.” (**H**) Co-localization of endogenous DDX54 and ALKBH5 in the nucleoplasm upon VSV infection. HEK293T cells were seeded in 6-well plates with cover clip overnight, followed by VSV infection (MOI = 1). Six hours later, cells were collected for immunofluorescence assays with DDX54 and ALKBH5 antibody. The results are representatives of at least three independent repeats.

A previous report showed that DDX54 is a nucleolar protein and is translocated to nucleoplasm upon ionizing radiation (20). Nucleus can be visualized by DAPI staining, showing a bright color in the nucleoplasm and a dark hole in the nucleolus (28, 29). A DDX54-fused GFP plasmid (DDX54-GFP) was overexpressed in HEK293T cells to illuminate the subcellular localization of DDX54. Compared with pEGFP-N3 that was ubiquitously expressed in the whole cells (Fig. 3A), overexpression of DDX54-GFP suggested that the strong GFP fluorescence signals accumulated in the nucleolus (Fig. 3B, top panels). However, poly(I:C) transfection or VSV infection enabled GFP signals to translocate from the nucleolus to the nucleoplasm (Fig. 3B, middle and bottom panels), which was verified by quantification of GFP signal (Fig. S1B). Tracking subcellular localization of endogenous DDX54 protein by a DDX54-specific antibody revealed similar results, showing that VSV infection facilitated DDX54 relocation from the nucleolus to the nucleoplasm (Fig. 3C).

### DDX54 binds to ALKBH5 in the nucleoplasm upon VSV infection

We hypothesized that DDX54 translocation upon immune stimulation enabled it to work with key enzymes involved in mRNA m^6^A modification. Co-IP assays revealed a constitutive interaction of ectopically expressed DDX54 with ALKBH5, a RNA m^6^A demethylase (8), and an enhanced interaction upon VSV infection (Fig. 3D) or poly(I:C) transfection (Fig. 3E). This interaction appeared to be enhanced in the presence of RNase (Fig. S1C). Using the antibody specific to DDX54 or ALKBH5, we also detected a constitutive interaction of endogenous DDX54 with ALKBH5 in resting cells, and an enhanced one in VSV-infected cells (Fig. 3F). Additionally, the association of DDX54 with METTL3 or METTL14, two m^6^A methyltransferases (7), was detected in resting cells or under poly(I:C) transfection (Fig. 3G), further indicating that DDX54 interacted with the cellular m^6^A machine in response to IFN stimuli. Unlike DDX54 largely accumulating in the nucleolus in resting cells, ALKBH5 predominately resided in the nucleoplasm, and under VSV infection, their co-localization was easily detected in the nucleoplasm (Fig. 3H). These results suggest that VSV infection facilitates the binding of DDX54 to ALKBH5 in the nucleoplasm.

### DDX54 promotes VSV replication and downregulates IFN response via ALKBH5

**Fig 4:**
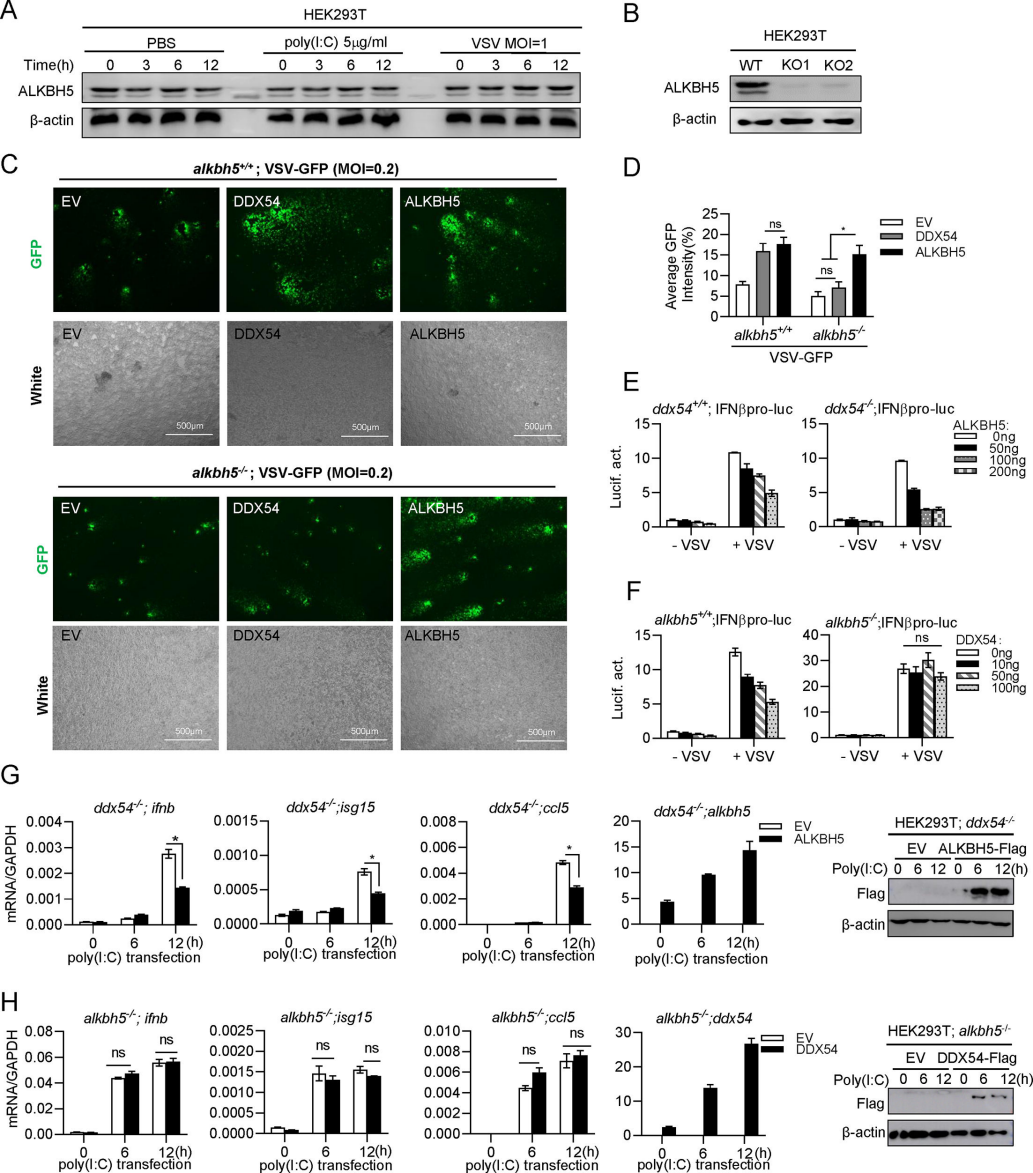
DDX54 promotes virus replication and downregulates IFN response via ALKBH5. (**A and B**) Western botting analyses of endogenous ALKBH5 protein in HEK293T cells following poly(I:C) transfection and VSV infection (**A**), or in *alkbh5+/+* and *alkbh5−*− HEK293T cells (**B**). The β-actin panel (**A**) is the same as the one shown in Fig. 2A indicating the samples were used in Fig. 2A and 4A. (**C and D**) DDX54 promoted virus replication via ALKBH5. *alkbh5^+/+^* and *alkbh5^−/^*^−^ cells seeded in 6-well plate overnight were transfected for 24 h with EV, DDX54, or ALKBH5 (1 µg each), followed by infection with VSV-GFP. At 12 h post infection, cells were visualized by fluorescence microscope (**C**) or subjected to evaluate virus replication by calculating the ratio of GFP positive/negative cells with software ImageJ (**D**). (**E and F**) DDX54 inhibited VSV-stimulated IFNβ protomer activation through ALKBH5. *ddx54^+/+^* and *ddx54^−/^*^−^ cells seeded in 48-well plates overnight were transfected with IFNβpro-luc (100 ng), pRL-TK (1 ng), together with increasing doses of ALKBH5 (**E**) or DDX54 (**F**). Twenty-four hours later, cells were infected with VSV (MOI = 1) for another 6 h, followed by luciferase assays. (**G and H**) DDX54 inhibited poly(I:C)-stimulated IFN response through ALKBH5. *ddx54^−/^*^−^ cells (**G**) or *alkbh5^−/^*^−^ cells (**H**) seeded in 12-well plates were transfected with ALKBH5 (**G**) or DDX54 (**H**) or with EV (500 ng each) as control. Twenty-four hours later, cells were transfected with poly(I:C) (5 µg/mL), followed by RT-qPCR analysis of *ifnb, isg15,* and *ccl5* at the indicated time points. Data were normalized to GAPDH. The overexpression of DDX54 protein was detected by western blotting analysis. Error bars represent SDs obtained by measuring each sample in triplicate. *P < 0.05, **P < 0.01, ***P < 0.001, Student *t* test. The results are representatives of at least three independent repeats.

We speculated that the interaction of DDX54 and ALKBH5 in nucleoplasm was required for DDX54 downregulating cellular IFN antiviral response. To this end, we sought to investigate the role of ALKBH5 in antiviral immunity by overexpression and knockout strategies. ALKBH5 protein was constitutively expressed in HEK293T cells, even under VSV infection or poly(I:C) transfection (Fig. 4A). CRISPR/Cas9 editing obtained ALKBH5-depleted HEK293T cells (*alkbh5^−/^*^−^) (Fig. 4B), which was verified by genomic DNA sequencing (Fig. S1D).

VSV-GFP infection revealed a weaker intensity of GFP signal (Fig. S2A and B), and a lower level of VSV genome RNAs in *alkbh5^−/^*^−^ cells than in *alkbh5^+/+^* cells (Fig. S2C). Additionally, overexpression of ALKBH5 in *alkbh5^+/+^* cells inhibited IFNβ promoter activation by VSV or poly(I:C), in a dose-dependent manner (Fig. S2D); compared with *alkbh5^+/+^* cells, *alkbh5^−/^*^−^ cells exhibited an enhanced IFNβ promoter activation by VSV infection (Fig. S2E). Similarly, VSV infection or poly(I:C) transfection upregulated mRNA expression of cellular *ifnb, isg15,* and *ccl5* more significantly in *alkbh5^−/^*^−^ cells than in *alkbh5^+/+^* cells (Fig. S2F). In *alkbh5^−/^*^−^ cells, ALKBH5 overexpression time-dependently downregulated these gene transcription by poly(I:C) transfection, compared with EV overexpression (Fig. S2G). These data suggest that like DDX54, ALKBH5 acts as an inhibitor of cellular IFN response to promote VSV replication, similar to a previous report (9).

We first determined the functional linking of DDX54 and ALKBH5 in virus replication. In *alkbh5^+/+^* cells, overexpression of either DDX54 or ALKBH5 yielded a relatively strong GFP fluorescence, compared with overexpression of empty vector (Fig. 4C, top panels). In *alkbh5^−/^*^−^ cells, DDX54 overexpression failed to promote VSV replication, but ALKBH5 overexpression restored VSV-GFP replication (Fig. 4C, bottom panels). Quantification of GFP intensities clearly indicated that DDX54 promoted VSV replication via ALKBH5 (Fig. 4D).

We next determined the functional linking of DDX54 and ALKBH5 in regulating the IFN response. In either *alkbh5^+/+^* cells or *alkbh5^−/^*^−^ cells, ALKBH5 overexpression significantly inhibited IFNβ promoter activation induced by VSV infection in a dose-dependent manner (Fig. 4E). However, DDX54 overexpression exhibited a similar inhibition just in *alkbh5^+/+^* cells but not in *alkbh5^−/^*^−^ cells (Fig. 4F). Similarly, the transcription induction of *ifnb, isg15,* and *ccl5* by poly(I:C) in *ddx54^−/^*^−^ cells was robustly inhibited by ALKBH5 (Fig. 4G), but not by DDX54 (Fig. 4H). Therefore, DDX54 downregulates cellular IFN antiviral response upstream of ALKBH5.

### DDX54 promotes the demethylase activity of ALKBH5 to erase m^6^A modification after VSV infection

**Fig 5:**
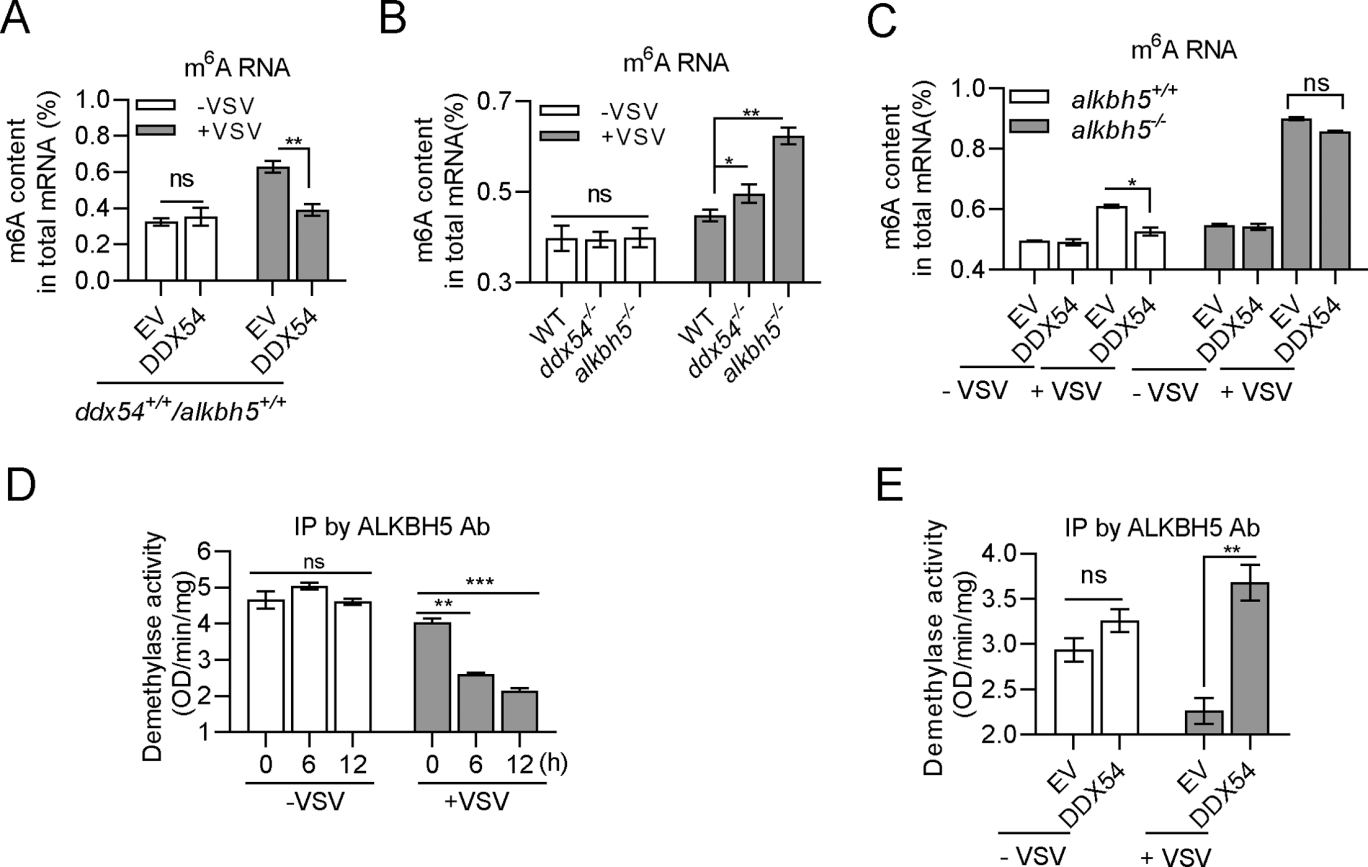
DDX54 promotes the enzymatic activity of ALKBH5 to erase m6A modification after VSV infection. (**A–C**) DDX54 decreased the abundance of m^6^A RNA via ALKBH5 after VSV infection. Wild-type (*alkbh5^+/+^*) HEK293T cells (**A**) or both *alkbh5^+/+^* and *alkbh5^−/^*^−^ cells (**C**) seeded in 6-well plate overnight were transfected for 24 h with EV and DDX54 (1 µg each), followed by VSV infection (MOI = 1). Or wild-type, *ddx54^−/^*^−^ and *alkbh5^−/^*^−^ cells were directed with VSV infection (MOI = 1) (**B**). Six hours later, cells were collected for total RNA extraction to detect m^6^A content with anti-m^6^A antibody. (**D and E**) DDX54 promoted m^6^A demethylation activity of ALKBH5 after VSV infection. HEK293T cells were seeded in 10 cm^2^ plates overnight followed by VSV infection (MOI = 1) for indicated times (**D**) or were transfected with ALKBH5, together with DDX54 or EV (5 µg each) for 24 h, followed by VSV infection (MOI = 1) for 8 h (E). Cells were collected for purification of ALKBH5 with anti-ALKBH5 Ab, followed by measurement of m^6^A demethylation activity. Error bars represent SDs obtained by measuring each sample in triplicate. *P < 0.05, **P < 0.01, ***P < 0.001, Student *t* test. The results are representatives of at least three independent repeats.

Given that ALKBH5 is a RNA m^6^A demethylase (8), we hypothesized that DDX54 might regulate mRNA metabolism to impair the IFN antiviral response via ALKBH5. Using the anti-m^6^A antibody, we immunoprecipitated a total of cellular m^6^A-modified RNAs (m^6^A RNAs) in WT HEK293T cells before and after VSV infection. It showed that VSV infection increased the abundance of m^6^A RNAs, which was significantly counteracted by DDX54 overexpression (Fig. 5A). Notably, DDX54 did not mediate the counteracting effect in the absence of VSV infection, likely due to the fact that DDX54 predominantly resides in the nucleolus in resting cells (Fig. 3B, C and H).

Despite similar abundance of m^6^A RNAs in uninfected WT cells, *ddx54^−/^*^−^ cells, and *alkbh5^−/^*^−^ cells, VSV infection yielded enhanced abundance of m^6^A RNAs, particularly in *ddx54^−/^*^−^ cells and *alkbh5^−/^*^−^ cells (Fig. 5B). Importantly, VSV infection-enhanced m^6^A modification was impaired by DDX54 overexpression in *alkbh5^+/+^* cells, but not in *alkbh5^−/^*^−^ cells (Fig. 5C), indicating that DDX54 reduces the amount of m^6^A RNAs through ALKBH5. We checked the enzymatic activity of cellular ALKBH5 in VSV-infected HEK293T cells. It showed that the demethylase activity of ALKBH5 was progressively decreased along with VSV infection (Fig. 5D), and at this time, DDX54 overexpression was able to restore the enzymatic activity of ALKBH5 (Fig. 5E). These results together indicate that VSV infection increases the amount of cellular m^6^A RNAs; however, DDX54 overexpression promotes the demethylase activity of ALKBH5, thus resulting in a decreased amount of m^6^A RNAs in VSV-infected cells.

### DDX54 and ALKBH5 independently bind to and interact with each other on selected m^6^A mRNAs after VSV infection

**Fig 6:**
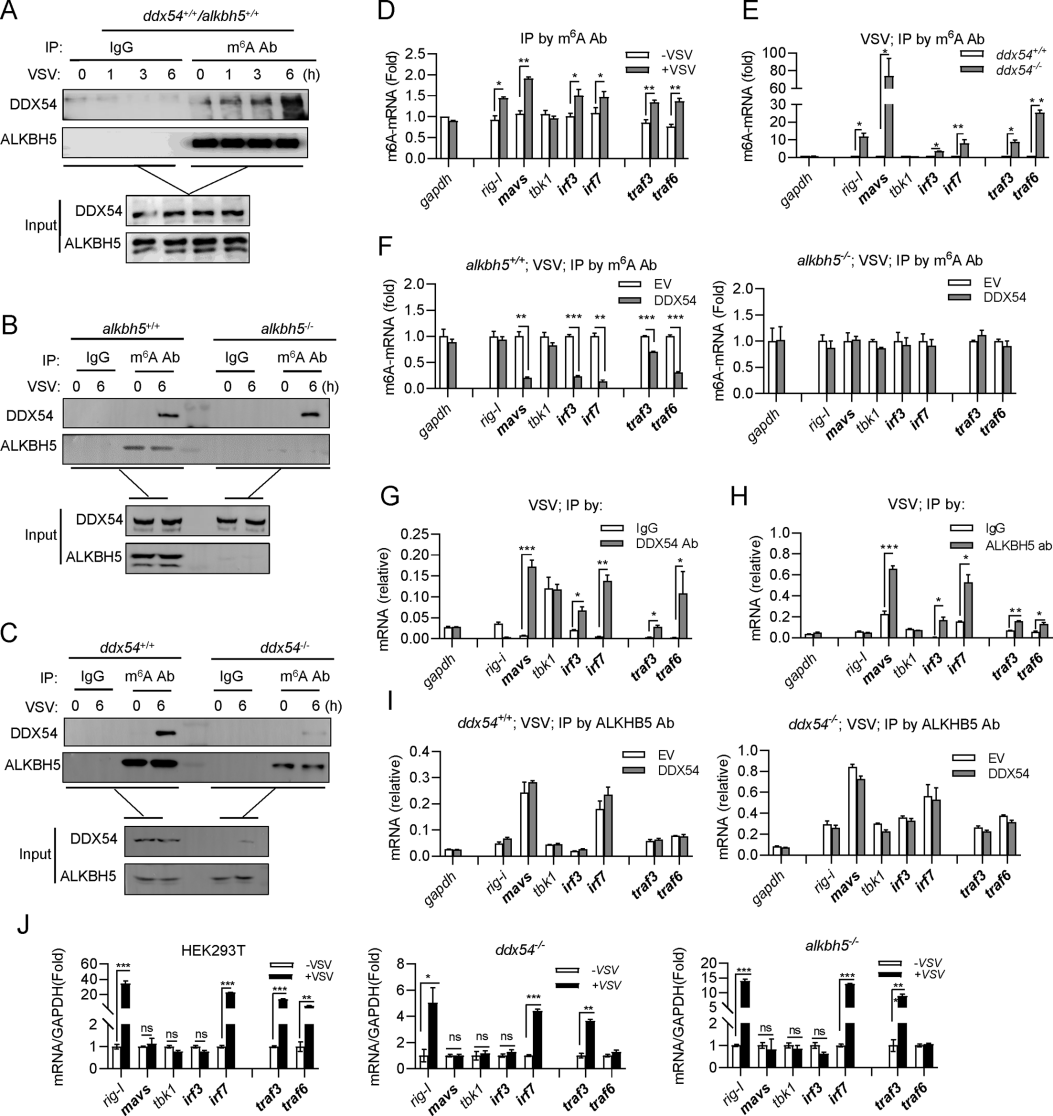
DDX54 and ALKBH5 independently bind to and interact with each other on selected m^6^A mRNAs after VSV infection. (**A–C**) DDX54 and ALKBH5 independently bound to m^6^A RNAs to form RNA/protein complexes upon VSV infection. HEK293T WT cells (**A**), together with *alkbh5^−/^*^−^ cells (**B**) or *ddx54^−/^*^−^ cells (**C**), were seeded in 10 cm^2^ plates overnight and were infected with VSV (MOI = 1). At the indicated times, cells were lysed for IP assays with anti-m^6^A Ab or anti-IgG Ab, followed by western blotting analyses of DDX54 and ALKBH5 protein in the immunoprecipitated RNA-protein complex. (**D–F**) DDX54 selectively decreased m^6^A modification through ALKBH5. HEK293T WT cells (**D**) or both *ddx54^+/+^* and *ddx54^−/^*^−^ cells (**E**) seeded in 3.5 cm^2^ plates overnight were directly infected with VSV (MOI = 1) for 6 h. Or *ddx54^+/+^* cells and *ddx54^−/^*^−^ cells were transfected with DDX54 (1 µg each) for 24 h, followed by infection with VSV for 6 h (**F**). Cell lysates were immunoprecipitated by anti-m^6^A Ab, followed by RT-PCR analyses. Data were normalized to GAPDH. (**G–I**) DDX54 and ALKBH5 independently bound to a similar subset of mRNAs upon VSV infection. *ddx54^+/+^* and *ddx54^−/^*^−^ cells seeded in 10 cm^2^ plates overnight cells were transfected for 24 h with DDX54 (1 µg) and subsequently infected for 6 h with VSV (MOI = 1) (**I**). Or *ddx54+/+* cells were directly infected with VSV for 6 h (**G and H**). Cell lysates were immunoprecipitated by anti-DDX54 Ab (**G**) or anti-ALKBH5 Ab (**H and I**), followed by RT-PCR analyses of mRNAs bound to DDX54 protein or ALKBH5 protein. Data were normalized to GAPDH. (**J**) RT-PCR analyses of gene expression in cells before and after VSV infection. HEK293T WT cells, *ddx54^−/^*^−^ and *alkbh5^−/^*^−^ cells seeded in 3.5 cm^2^ plates overnight were directly infected with or without VSV (MOI = 1) for 6 h. Cells were harvested for RT-PCR analyses. Error bars represent SDs obtained by measuring each sample in triplicate. *P < 0.05, **P < 0.01, ***P < 0.001, Student *t* test. The results are representatives of at least three independent repeats.

To determine how DDX54 or ALKBH5 impacted m^6^A RNAs upon VSV infection, we used the anti-m^6^A antibody to immunoprecipitate m^6^A-marked RNA/protein complexes for western blotting analyses of DDX54 or ALKBH5 in the complex. It showed that DDX54 progressively bound to m^6^A RNAs upon VSV infection, but the binding of ALKBH5 to m^6^A RNAs remained constant in WT cells (*ddx54^+/+^*/*alkbh5^+/+^*) (Fig. 6A). The formation of m^6^A RNAs/protein complexes highlights an interaction of DDX54 with ALKBH5 on cellular m^6^A RNAs. In *alkbh5^−/^*^−^ cells, DDX54 still bound to m^6^A RNAs under virus infection (Fig. 6B), and in *ddx54^−/^*^−^ cells, a constant binding was also seen for ALKBH5 regardless of virus infection (Fig. 6C), indicating that in response to VSV infection, DDX54 recognizes and binds to the m^6^A RNAs independently of ALKBH5, and vice versa.

Given that VSV infection triggers cellular IFN signaling by the RLR pathway (11), we hypothesized that DDX54 worked with ALKBH5 on m^6^A mRNAs of RLR signaling factors (*rig-i,mavs, tbk1, irf3. irf7*) to impair IFN response. To this end, we used the anti-m^6^A antibody to immunoprecipitate m^6^A-marked RNA/protein complexes for qPCR analysis of m^6^A modification on the above-mentioned mRNAs. Expectedly, VSV infection increased the abundance of m^6^A-modified mRNAs of *rig-i, mavs, irf3,* and *irf7* but not of *tbk1* in WT cells (Fig. 6D), and these mRNAs displayed higher levels of m^6^A modification in *ddx54^−/^*^−^ cells than in *ddx54^+/+^* cells (Fig. 6E). Both *traf3* and *traf6* were included as positive control and *tbk1* as negative control because a previous report showed that both *traf3* and *traf6* mRNAs rather than *tbk1* mRNA can be m^6^A-modified by VSV infection (9). These results suggest that DDX54 selectively binds to some m^6^A-marked transcripts upon VSV infection. Additionally, DDX54 overexpression downregulated the m^6^A-modified mRNAs of *mavs, irf3, irf7, traf3,* and *traf6* but not of *tbk1* and *rig-i* in *alkbh5^+/+^* cells, and this downregulation was not seen in *alkbh5^−/^*^−^ cells (Fig. 6F), indicating that DDX54 selectively decreases VSV infection-induced m^6^A modification on *mavs, irf3, irf7, traf3,* and *traf6* mRNAs via ALKBH5.

In VSV-infected WT cells, RIP assays by using DDX54 antibody showed that DDX54 selectively bound to mRNAs of *mavs, irf3, irf7, traf3,* and *traf6,* but not of *tbk1* and *rig-i* (Fig. 6G). Using the ALKBH5 antibody, similar binding profiles of ALKBH5 were also detected upon virus infection (Fig. 6H). These results indicate that both DDX54 and ALKBH5 selectively bind to a common subset of targeted mRNAs. However, DDX54 overexpression did not make any differences on ALKBH5 binding to these transcripts in *ddx54^+/+^* cells or *ddx54^−/^*^−^ cells (Fig. 6I).

Finally, we compared the transcription expression of these selected genes in WT cells, *ddx54^−/^*^−^ cells, and *alkbh5^−/^*^−^ cells before and after VSV infection (Fig. 6J). It showed that virus infection did not alter the transcription levels of *mavs, irf3,* and *tbk1* in WT cells, *ddx54^−/^*^−^ cells, and *alkbh5^−/^*^−^ cells. Considering that VSV increased m^6^A methylation of *mavs* and *irf3* mRNAs but not of *tbk1* mRNA, these results excluded the possibility that the increased m^6^A modification on these selected transcripts, at least on *mavs* and *irf3* mRNAs, is related to the virus-induced transcriptional upregulation. Taken together, these data suggest that VSV infection drives either DDX54 or ALKBH5 to independently and selectively bind to a common subset of m^6^A-marked transcripts, on which DDX54 interacts with and promotes the enzymatic activity of ALKBH5, finally demethylating these transcripts.

### DDX54 retains *mavs* mRNA in the nucleus to downregulate antiviral response

**Fig 7:**
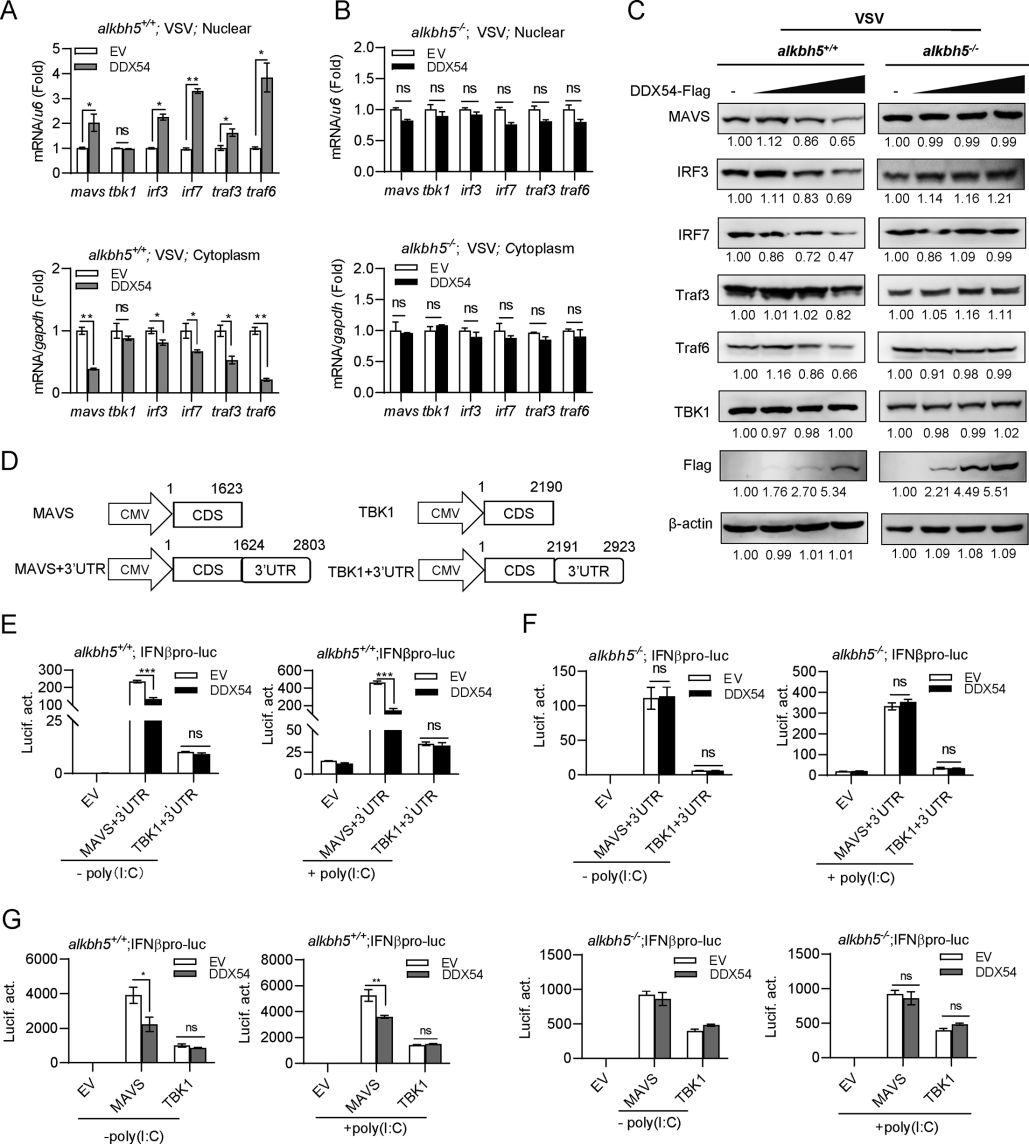
DDX54 retains *mavs* mRNA in the nucleus to downregulate antiviral response. (**A and B**) DDX54 facilitated mRNA nuclear retention through ALKBH5. *alkbh5^+/+^* cells (**A**) and *alkbh5^−/^*^−^ cells (**B**) seeded in 10 cm^2^ plates overnight were transfected with EV or DDX54 (5 µg each) plasmid for 24 h and subsequently infected for 6 h with VSV (MOI = 1). Cells were harvested to separate nuclear RNA and cytoplasmic RNA, followed by RT-PCR analyses of the indicated mRNAs. (**C**) DDX54 attenuated protein expression of selected transcripts through ALKBH5. *alkbh5^+/+^* cells and *alkbh5^−/^*^−^ cells seeded in 12-well plates overnight were transfected for 24 h with DDX54-Flag plasmid at increasing dose (0, 0.1, 0.5, 1 µg) and subsequently infected for 6 h with VSV infection (MOI = 1), followed by western blotting. The numbers show the densitometric quantification of protein expression. (**D**) Schematic diagrams of plasmids MAVS + 3′UTR, TBK1 + 3′UTR. (**E and F**) DDX54 inhibited IFNβ promoter activation by MAVS + 3’UTR but not by TBK1 + 3’UTR. *alkbh5^+/+^* cells (**E**) and *alkbh5^−/^*^−^ cells (**F**) seeded in 48-well plates overnight were transfected with IFNβpro-luc (100 ng), pRL-TK (1 ng), DDX54 or EV (100 ng each), together with MAVS + 3′UTR or TBK1 + 3′UTR (100 ng each). Twenty-four hours later, cells were transfected with or without poly(I:C) (5 µg/mL). Another 6 h later, cells were harvested for luciferase assays. Error bars represent SDs obtained by measuring each sample in triplicate. *P < 0.05, **P < 0.01, ***P < 0.001, Student *t* test. The results are representatives of two independent repeats. (G) DDX54 inhibited IFNβ promoter activation by MAVS but not by TBK1. Cell transfection was performed as described for panels E and F using MAVS and TBK1 plasmids.

We wondered whether the DDX54/ m^6^A/ALKBH5 axis impaired the nuclear export and translation of the targeted m^6^A mRNAs. To this end, we fractioned cellular nucleus and cytoplasm. It showed that in *alkbh5^+/+^* cells, DDX54 overexpression resulted in a robust nuclear accumulation of *mavs, irf3, irf7, traf3,* and *traf6* mRNAs, all of which were concomitantly attenuated in the cytoplasm (Fig. 7A). In *alkbh5^−/^*^−^ cells, mRNA nuclear accumulation and the following cytoplasmic attenuation were not seen (Fig. 7B). These results indicate that DDX54 is able to retain *mavs, irf3, irf7, traf3,* and *traf6* mRNAs in the nucleus, except for *tbk1* mRNA. Consistently, DDX54 overexpression dose-dependently reduced protein expression of MAVS, IRF3, IRF7, TRAF3, and TRAF6 but not of TBK1 in *alkbh5^+/+^* cells, which was not detected in *alkbh5^−/^*^−^ cells (Fig. 7C). Similar results were seen when protein expression was compared between *ddx54^−/^*^−^ cells and WT cells (Fig. S3).

Subsequently, we took *mavs* mRNA as an example to test whether DDX54 was able to inhibit MAVS-triggered IFN response through the ALKBH5-mediated demethylation. The m^6^A methylation occurs mainly in 3′UTRs and near stop codons (30), and in mouse, the m^6^A modification is indeed present in the 3′UTR and the CDS adjacent to the stop codon of *mavs* mRNA but not in *tbk1* mRNA (14). We generated a MAVS expression plasmid by cloning its ORF alone or together with 3′UTR into pcDNA3.1 (MAVS + 3′UTR) (Fig. 7D). In *alkbh5^+/+^* cells, overexpression of MAVS + 3′UTR plasmid could stimulate IFNβ promoter activity, and this stimulation was effectively inhibited by DDX54 overexpression (Fig. 7E). In *alkbh5^−/^*^−^ cells, no inhibitory effect was detected (Fig. 7F). As control, TBK1 + 3′UTR plasmid-triggered IFNβ promoter activation was not impaired by DDX54 in either *alkbh5^+/+^* cells or *alkbh5^−/^*^−^ cells (Fig. 7E and F). We repeated these assays using MAVS plasmid, and the same results were obtained (Fig. 7G). Therefore, the 3′UTR of *mavs* does not have any influence on the inhibitory effect of DDX54. However, these results further suggest that DDX54 selectively targets some transcripts, such as *mavs* mRNA, to inhibit IFN antiviral response via ALKBH5.

### The ATPase activity of DDX54 is essential for its downregulating IFN antiviral response

**Fig 8:**
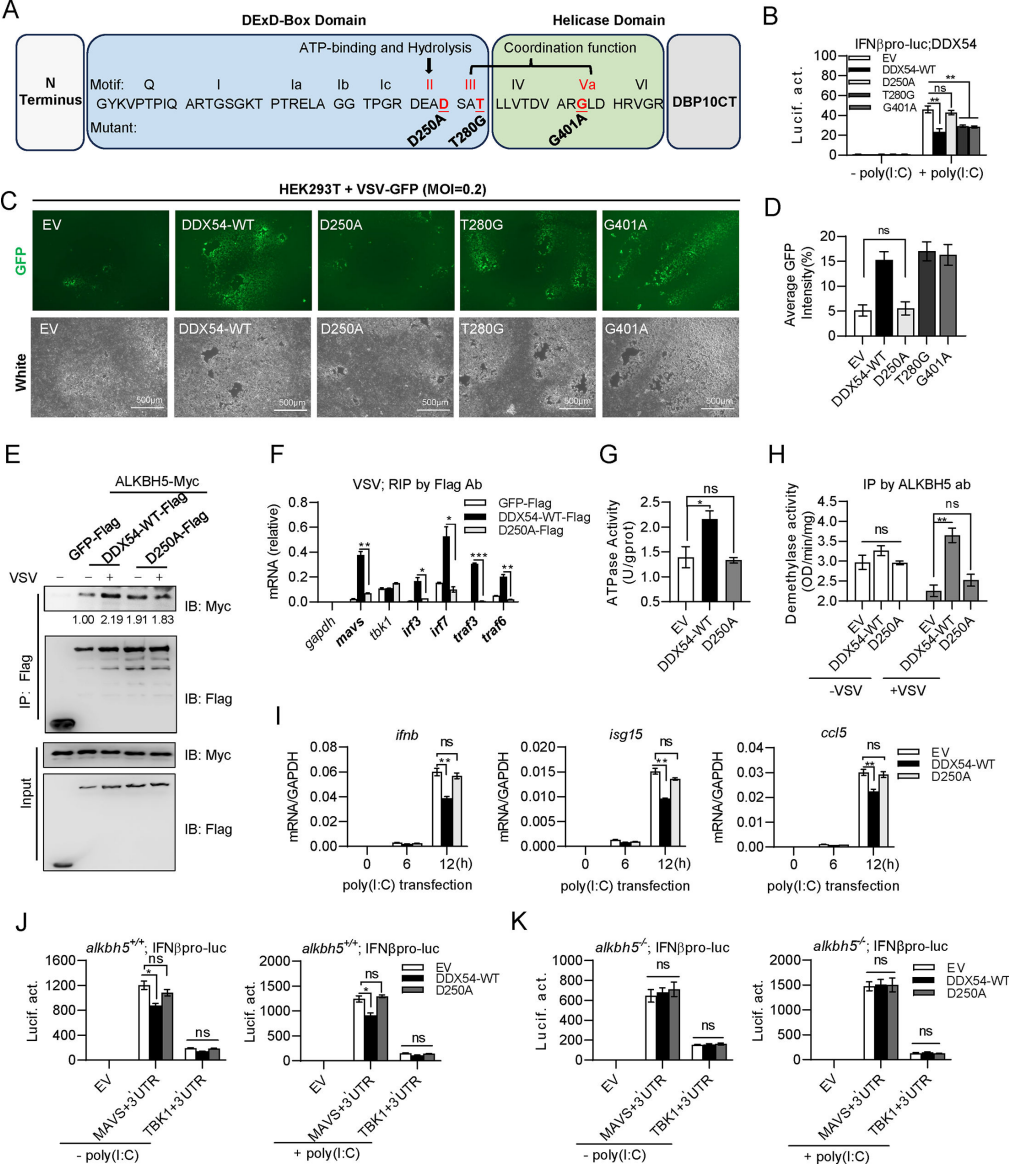
The ATPase activity of DDX54 is essential for its downregulating IFN antiviral response. Schematic diagrams of DDX54 point mutants (D250A, T280G, and G401A). D250A failed to inhibit IFNb promoter activation by VSV infection. HEK293T cells seeded in 48-well plates overnight were transfected for 24 h with IFNβpro-luc (100 ng), pRL-TK (1 ng), together with DDX54 or each mutant (100 ng each), followed by infection with VSV (MOI = 1) for 6 h. (**C and D**) D250A failed to promote VSV-GFP replication. HEK293T cells seeded in 6-well plates were transfected for 24 h with DDX54 or each mutant (100 ng each), followed by infection with VSV-GFP for 12 h. Cells were visualized by fluorescence microscope (**C**), or subjected to evaluate virus replication by calculating GFP-positive cells with software ImageJ (**D**). (**E**) D250A interaction with ALKBH5 was not enhanced by VSV infection. HEK293T cells seeded in 10 cm^2^ plates overnight were transfected for 24 h with the indicated plasmids (5 µg each), followed by infection with or without VSV (MOI = 1). Six hours later, cells were harvested for Co-IP assays with tag antibodies. (**F**) D250A failed to selectively bind mRNAs upon VSV infection. HEK293T cells seeded in 3.5 cm^2^ plates overnight were individually transfected for 24 h with the indicated plasmids (1 µg each), followed by infection with VSV (MOI = 1). Another 6 h later, cells were collected for RIP assays. Data were normalized to GAPDH. (**G**) D250A lost ATPase activity. HEK293T cells seeded in 10 cm^2^ plates overnight were individually transfected with the indicated plasmids (10 µg each). Thirty-six hours later, cells were collected for extraction of nuclear protein, which was subjected to measure ATPase activity. (**H**) D250A failed to promote the enzymatic activity of ALKBH5. HEK293T cells seeded in 10 cm^2^ plates overnight were individually transfected with the indicated plasmids (5 µg each) for 24 h, followed by VSV infection (MOI = 1) for 8 h. Cells were collected for purification of ALKBH5 with anti-ALKBH5 Ab, followed by measurement of m^6^A demethylation activity. (**I**) D250A failed to inhibit IFN response. HEK293T cells seeded in 12-well plates were individually transfected for 24 h with the indicated plasmids (500 ng each), followed by infection with VSV (MOI = 1). Six hours later, cells were collected for RT-PCR analysis. Data were normalized to GAPDH. (**J and K**) D250A failed to inhibit IFNβ promoter activation by MAVS + 3′UTR. *alkbh5+/+* cells (**J**) and *alkbh5−/*− cells (**K**) seeded in 48-well plates overnight were transfected with IFNβpro-luc (100 ng), pRL-TK (1 ng), DDX54 or D250A, together with MAVS + 3′UTR or TBK1 + 3′UTR (100 ng each). Twenty-four hours later, cells were transfected with or without poly(I:C) (5 µg/mL). Another 6 h later, cells were harvested for luciferase assays. Error bars represent SDs obtained by measuring each sample in triplicate. *P < 0.05, **P < 0.01, ***P < 0.001, Student *t* test. The results are representatives of at least three independent repeats.

DDX54 is a typical ATP-dependent RNA helicase (31), with eight conserved motifs (Fig. S4A), whose function has been well-characterized in RNA helicases (17, 18). Based on multiple alignments of DDX54 with other RNA helicases (Fig. S4B), we generated three mutants of DDX54, one bearing mutation in ATPase activity (D250A) and two in ATP hydrolysis and nucleic acid binding (T280G, G401A) (Fig. 8A). Similar mutations have been verified in DDX46 (9), DHX16 (32), and DDX39A (16).

Luciferase assays showed that overexpression of D250A rather than T280G or G401A failed to downregulate IFNβ promoter activity induced by poly(I:C) (Fig. 8B). Consistently, overexpression of D250A failed to promote VSV-GFP replication, while T280G and G401A exhibited a similar function to DDX54-WT (Fig. 8C and D). Hence, DDX54-mediated downregulation is dependent on the ATP-binding DEAD motif but not SAT or ARGLD motifs.

We further sought to determine how D250A lost the potential to inhibit IFN antiviral response. In initial experiments, immunofluorescence observations showed that like DDX54-WT, VSV infection drove D250A to translocate from the nucleolus to the nucleoplasm and subsequently interacted with ALKBH5 in the nucleoplasm (Fig. S5A). Further Co-IP assays showed a constitutive binding of D250A to ALKBH5, as did DDX54-WT (Fig. S5B).

However, unlike DDX54-WT showing an enhanced interaction with ALKBH5 upon VSV infection, D250A exhibited a similar binding to ALKBH5 regardless of VSV infection (Fig. 8E). RIP assays showed that unlike DDX54-WT that could increasingly bind to the selected mRNAs upon VSV infection, D250A did not increasingly bind to mRNAs of *mavs, irf3,* and *irf7* after VSV infection (Fig. 8F). Indeed, D250A lost the intact ATPase activity (Fig. 8G), failed to promote the enzymatic activity of ALKBH5 during VSV infection (Fig. 8H), and thus failed to inhibit poly(I:C)-induced mRNA expression of *ifnb, isg15,* and *ccl5* (Fig. 8I). Consistently, unlike DDX54-WT, D250A failed to inhibit MAVS + 3′UTR plasmid-induced IFNβ promoter activation, either in *alkbh5^+/+^* cells (Fig. 8J) or in *alkbh5^−/^*^−^ cells (Fig. 8K). These results indicate that DDX54 selectively binds to the targeted transcripts and promotes the demethylation activity of ALKBH5 dependent on its intact ATPase activity.

## DISCUSSION

RNA helicases are often believed to participate in the RNA unwinding; however, only a subset displays a processive helicase function because others show a broad array of biochemical functions, such as in the innate antiviral immune response (17, 18). For example, DDX42 functions as a direct effector to selectively inhibit the replication of positive RNA viruses by its RNA binding potential (33); DDX39A specifically controls alphavirus by recognizing a conserved viral RNA structural element (34). Three RLR receptors (RIG-I, MDA5, and LGP2, belonging to DExD/H-box helicase family), DHX16, and DDX1/DDX21/DHX36 complex function as viral dsRNA sensors to trigger host IFN response (35–37). Additionally, DDX24, DDX46, and DDX5 have a potential to fine tune the IFN response (9, 16, 38). In this study, we identify DDX54 as a negative regulator of cellular IFN antiviral response. Mechanistically, VSV infection enables DDX54 to translocate from the nucleolus to the nucleoplasm, where DDX54 interacts with ALKBH5 to demethylate the m^6^A modification on the selected transcripts, including *mavs*, *irf3/7,* and *traf3/6* mRNAs. This demethylation results in nuclear retention and translation restriction of these transcripts, finally impairing cellular IFN response. Our results provide an insight into how a DDX54/m^6^A/ALKBH5 axis fine tunes cellular antiviral response.

We provide evidence that DDX54 downregulates host IFN response and concomitantly promotes VSV replication by working with ALKBH5 to impair the m^6^A modification on selected transcripts. First, VSV infection induces an increasing interaction of DDX54 with the components of m^6^A machinery (including ALKBH5, METTL3, and METTL14), and also with m^6^A mRNAs. Second, individual and combinatory gene function analyses reveal that DDX54 downregulates cellular IFN antiviral response upstream of ALKBH5. Third, in VSV-infected cells, DDX54 decreases cellular abundance of m^6^A RNAs, such as m^6^A-modified mRNAs of *mavs*, *irf3/7,* and *traf3/6*, which are strictly dependent on ALKBH5. Finally, given that *mavs* mRNA rather than *tbk-1* mRNA is precisely demethylated by DDX54 working with ALKBH5, DDX54 can inhibit MAVS-triggered but not TBK1-triggered IFN antiviral response in *alkbh5^+/+^* cells but not in *alkbh5^−/^*^−^ cells. These results together indicate that DDX54 is required to interact with ALKBH5 and subsequently promotes the demethylation of the selected m^6^A-modified transcripts, for example, *mavs* mRNA rather than *tbk-1* mRNA, to suppress cellular IFN antiviral response.

Our results highlight that DDX54 relocation from the nucleolus to the nucleoplasm is associated with its inhibitory function. In resting cells, DDX54 is largely expressed in the nucleolus, a primary site where rRNAs are transcribed, processed, and assembled with ribosomal proteins (39), so it is believed to initiate peptidyl transferase center formation during the biogenesis of large (60S) ribosomal subunits (19). In the current study, VSV infection facilitates DDX54 to relocate to the nucleoplasm, a central area where all mRNAs are transcribed and processed. These findings indicate that DDX54, as a typical RNA helicase, might interact with different types of nucleic acids, depending on its distinct subcellular localization and in response to different cellular stresses (19, 20, 40). VSV infection-driven relocation enables DDX54 to bind to some mRNAs with m^6^A methylation, such as *mavs*, *irf3/7,* and *traf3/6* mRNAs, which is essential for DDX54 to downregulate the IFN antiviral response by promoting ALKBH5-mediated demethylation and translation restriction of these transcripts. Nucleoplasm relocation of DDX54 is also seen in human breast carcinoma cells exposed to ionizing radiation (IR); however, this relocation enables DDX54 to facilitate alternative splicing of IR-induced pre-mRNAs (20). Therefore, under different cellular stresses, DDX54 might target different RNA substrates to exert different physiological functions.

We attempt to clarify how DDX54 synergizes with ALKBH5 to selectively demethylate transcripts for host factors involved in antiviral signaling. We first provide evidence that VSV infection drives the formation of RNA/protein complexes that are composed of DDX54, ALKBH5, and cellular m^6^A RNAs. Combined with the findings that DDX54 progressively binds to cellular m^6^A mRNAs and also increasingly interacts with ALKBH5 just under VSV infection, these results together imply that there is an increasing interaction of DDX54 with ALKBH5 on cellular m^6^A RNAs after VSV infection. In addition, VSV infection facilitates DDX54 to bind to the m^6^A-modified mRNAs of *mavs*, *irf3/7,* and *traf3/6* but not of *tbk1*, highlighting a selective binding of DDX54 to these m^6^A-modified transcripts upon VSV infection. Despite that ALKBH5 displays a constant binding amount of cellular m^6^A RNAs regardless of virus infection, VSV infection still facilitates ALKBH5 to bind to the selected m^6^A RNAs, including *mavs*, *irf3/7,* and *traf3/6* mRNAs but not of *tbk1*mRNA. Importantly, during VSV infection, either DDX54 or ALKBH5 can bind to m^6^A RNAs independently of each other, and ALKBH5 still increasingly binds to *mavs*, *irf3/7,* and *traf3/6*mRNAs but not to *tbk1* mRNA in *ddx54^−/^*^−^ cells, as it does in *ddx54^+/+^* cells. These results further demonstrate that in response to VSV infection, DDX54 and ALKBH5 can independently and increasingly bind to a common subset of selected m^6^A-modified transcripts, on which DDX54 and ALKBH5 interact with each other.

We think that the interaction of DDX54 and ALKBH5 on the selected m^6^A-modified transcripts is essential for subsequent demethylation of these selected transcripts. We further provide evidence that VSV infection generally impairs the total demethylase activity of cellular ALKBH5, a finding similar to a previous report (41), and instead, DDX54 overexpression can effectively promote the demethylase activity of ALKBH5. Therefore, it is easy to understand why VSV infection generally increases the total amounts of cellular m^6^A RNAs in the virus-infected cells, which is, at least partially, due to the impaired demethylase activity of ALKBH5. On the contrary, the m^6^A modification on the selected transcripts is decreased. This is because VSV infection-directed interaction of DDX54 and ALKBH5 on the selected transcripts provides a platform for DDX54 to help restore the demethylase activity of ALKBH5, and thus ALKBH5, in turn, facilitates the demethylation of the selected transcripts.

Consistent with the above findings, the ATPase-deficient DDX54 mutant D250A fails to inhibit cellular IFN antiviral response, probably because it loses the ability to restore the demethylase activity of ALKBH5 on the selected mRNAs. In the absence of VSV infection, DDX54 does not bind to cellular m^6^A RNAs and, thus, has no effect on the enzymatic activity of ALKBH5, as evidenced by the fact that depletion of either DDX54 or ALKBH5 indeed does not affect the abundance of cellular m^6^A RNAs in resting cells. These results further support the notions that DDX54-driven ALKBH5 demethylation of the selected transcripts occurs just under VSV infection and that the interaction of DDX54 and ALKBH5 on the selected transcripts is essential for restoring enzyme activity of ALKBH5, subsequently demethylating the selected transcripts, and finally downregulating the IFN antiviral response.

Therefore, it is unlikely that DDX54 can interact with ALKBH5 on cellular m^6^A RNAs in the absence of VSV infection because DDX54 predominately locates to the nucleolus. Interestingly, a constitutive interaction of DDX54 with ALKBH5 is detected by Co-IP assays. We think that this constitutive interaction might be due to a fact that there is a very low level of DDX54 protein in the nucleoplasm of resting cells because all nucleolar components are highly dynamic, often exhibiting continual flux within the nucleolus and dynamic exchange with the surrounding nucleoplasm (39). Given that DDX54 does not bind to the m^6^A-modified mRNAs in resting cells, it is obvious that this constitutive interaction might not contribute to regulating the IFN signaling by a similar mechanism.

In response to VSV infection, ALKBH5 protein expression and subcellular localization are not influenced, which are also seen in a previous report (41). However, ALKBH5 is redistributed to the cytoplasm upon herpes simplex virus (HSV-1) infection, which is accompanied by a wide-scale reduction in the installation of m^6^A and other RNA modifications on host and viral mRNAs (42). Despite different subcellular localization of ALKBH5 in response to different virus infection, depletion of m^6^A machinery including ALKBH5 generally leads to enhanced IFN antiviral response by regulation of the m^6^A RNA abundances (9–13, 42). This means a complex regulation of cellular IFN antiviral response through ALKBH5, likely because different cellular mechanisms are activated in response to different viruses. This variation might be dependent on the sensitivity of a given virus to type I IFNs, the expression of m^6^A machinery components, and the integrated effects of m^6^A modification on both host and viral mRNAs. Although our results have shown that DDX54 is involved in the demethylation of selected transcripts, a comprehensive profiling of DDX54-driven change in the installation of m^6^A on cellular and VSV RNAs is waiting for further clarification.

Taken together, our results suggest that DDX54 is a unique m^6^A RNA-binding protein that has a potential to regulate ALKBH5 demethylase activity on the selected m^6^A mRNA targets to benefit VSV replication. A current report shows that RBM33 guides a subset of mRNA m^6^A demethylation by activating ALKBH5 demethylase activity through the removal of ALKBH5’s SUMOylation (43). How DDX54 activates ALKBH5 demethylase activity is another interesting issue in the future.

## Data Availability

All relevant data are within the paper and its supplemental material.

## SUPPLEMENTAL MATERIAL

Supplemental material jvi.00507-25-s0001.pdf Figures S1 to S5 and Table S1.

ASM does not own the copyrights to Supplemental Material that may be linked to, or accessed through, an article. The authors have granted ASM a non-exclusive, world-wide license to publish the Supplemental Material files. Please contact the corresponding author directly for reuse.
