# Supplementary material for: DDX54 drives ALKBH5-mediated demethylation of selected transcripts to suppress interferon antiviral response
Source: J Virol. 2025 Aug 12;99(9):e00507-25. doi: 10.1128/jvi.00507-25 (PMC12456001; doi:10.1128/jvi.00507-25)
Supplement: Supplemental material — Figures S1 to S5 and Table S1. [file jvi.00507-25-s0001.pdf]

Supplementary Figures

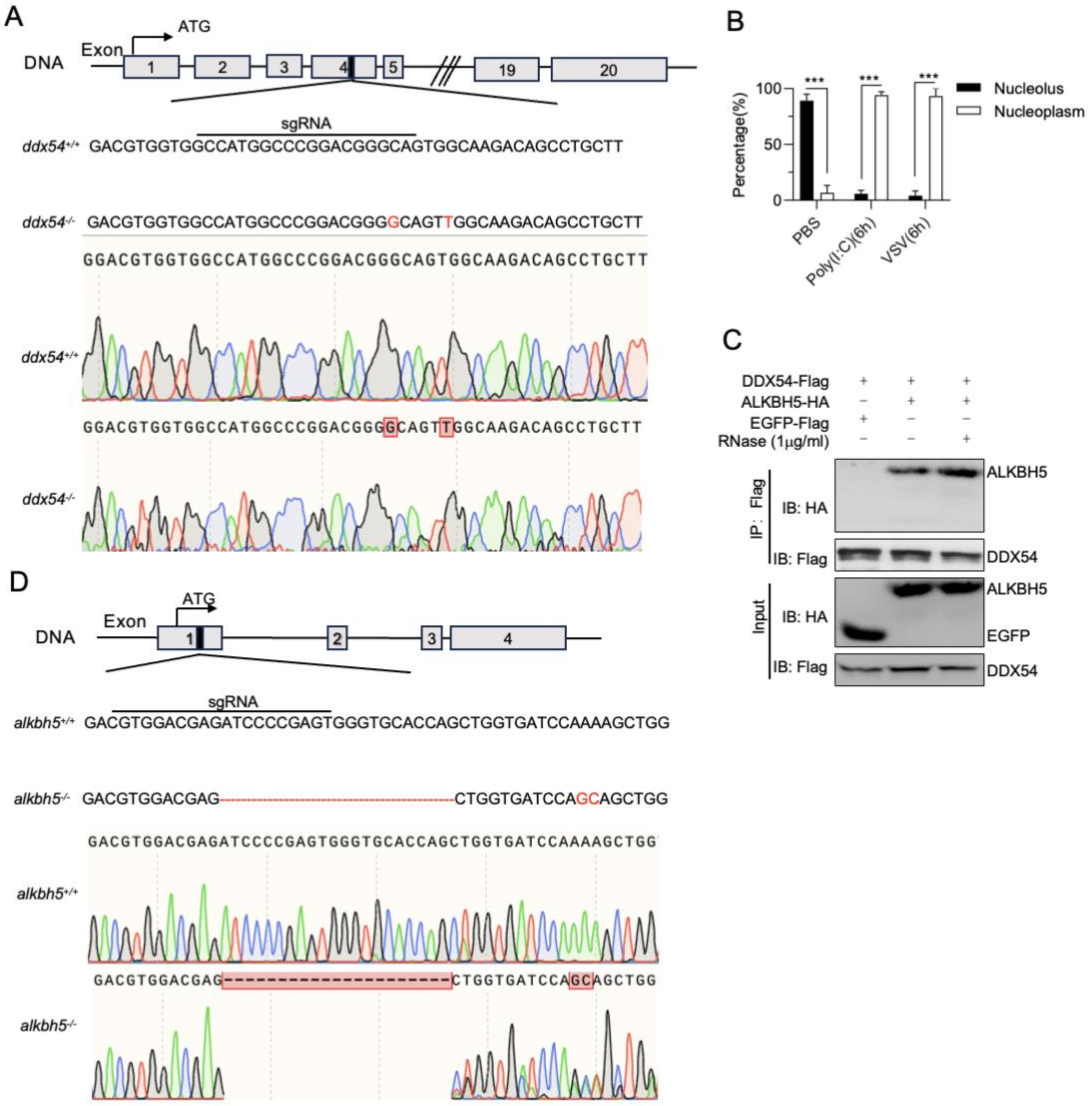

Supplementary Fig.1. Genomic analyses of knockout cells and the interaction of DDX54 and ALKBH5.

(A) Schematic of DDX54 genomic gene shows the CRISPR/Cas9 targeting site.

(B)The quantification of DDX54 relocalization from the nucleolus to the nucleoplasm.

(C) The interaction of DDX54 and ALKBH5. HEK293T cells seeded in 10 cm^2 plates overnight were transfected for 24h with DDX54-Flag and ALKBH5-Flag (5μg each). 6h later, cells were collected for Co-IP assays in the presence of RNase with tag-specific antibody.

(D) Schematic of ALKBH5 genomic gene shows the CRISPR/Cas9 targeting site.

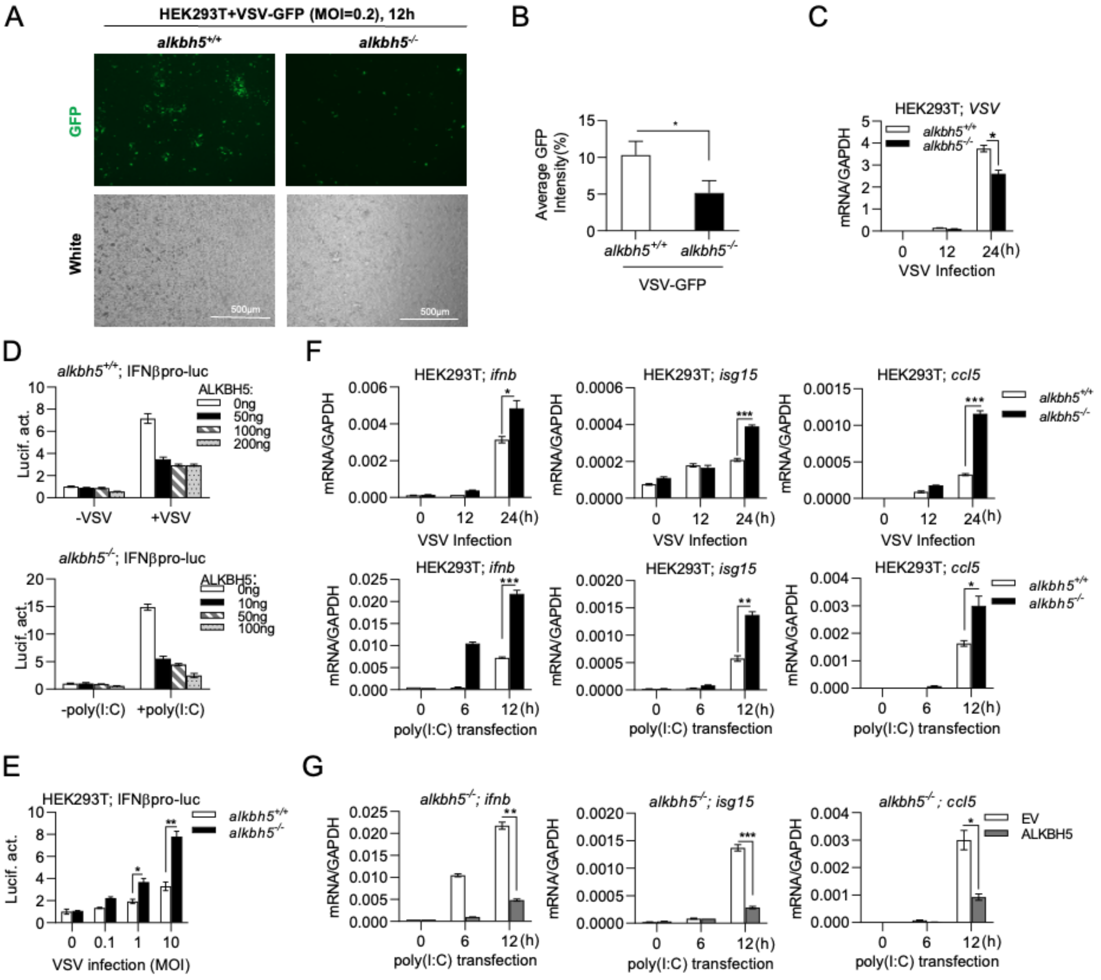

Supplementary Fig.2. Knockout of ALKBH5 inhibits virus replication and enhances IFN response.

(A-C) Knockout of ALKBH5 inhibited VSV replication. *alkbh5*$^{+/+}$ and *alkbh5*$^{-/-}$ HEK293T cells seeded in 6-well plate overnight were infected with VSV-GFP (A and B) or VSV (C, MOI=1). At 12 h post infection, cells were visualized by fluorescence microscope (A) or subjected to evaluate virus replication by calculating GFP-positive cells with software ImageJ (B). Or cells were collected for RT-PCR analyses of VSV genome RNA at the indicated times (C)

(D-E) ALKBH5 inhibited IFNβ protomer activation by VSV or poly(I:C). *alkbh5*$^{+/+}$ HEK293T cells (D) or *alkbh5*$^{+/+}$ and *alkbh5*$^{-/-}$ cells (E) seeded in 48-well plates overnight were transfected with IFNβpro-luc (100ng) and pRL-TK (1ng) (E),

together with ALKBH5 at increasing doses (D). 24h later, cells were infected with VSV at MOI=1 or poly(I:C) at 5μg/ml (D) or with VSV at increasing doses (MOI=0, 0.1, 1 and 10). Another 6h later, cells were harvested for luciferase assays.

(F-G) ALKBH5 inhibited IFN response by VSV or poly(I:C). Both *alkbh5*$^{+/+}$ and *alkbh5*$^{-/-}$ cells seeded in 12-well plates overnight were infected with VSV (MOI=1) or transfection with poly(I:C) (5μg/ml) (H). Or *alkbh5*$^{-/-}$ cells were transfected with EV or ALKBH5 (500ng each) for 24h, followed by infection with VSV (MOI=1) (I). Cells were collected at the indicated time points for RT-qPCR analysis of *ifnb, isg15 and ccl5.* Data were normalized to GAPDH.

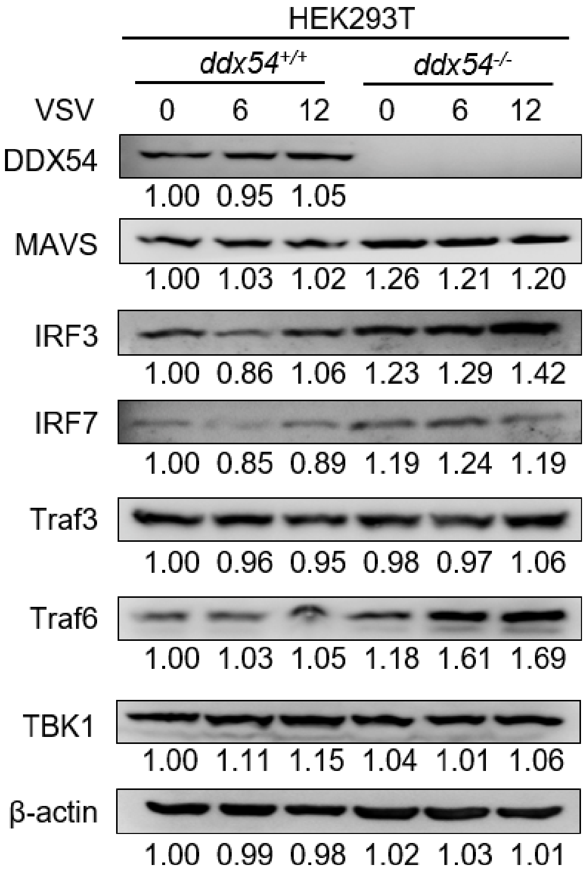

Supplementary Fig.3. DDX54 attenuated protein expression of selected antiviral transcripts through ALKBH5. WT cells and *ddx54*$^{-/-}$ cells seeded in 12-well plates overnight were infected with VSV infection (MOI=1). At the indicated time points, cells were harvested for western blotting. The numbers show the densitometric quantification of protein expression.

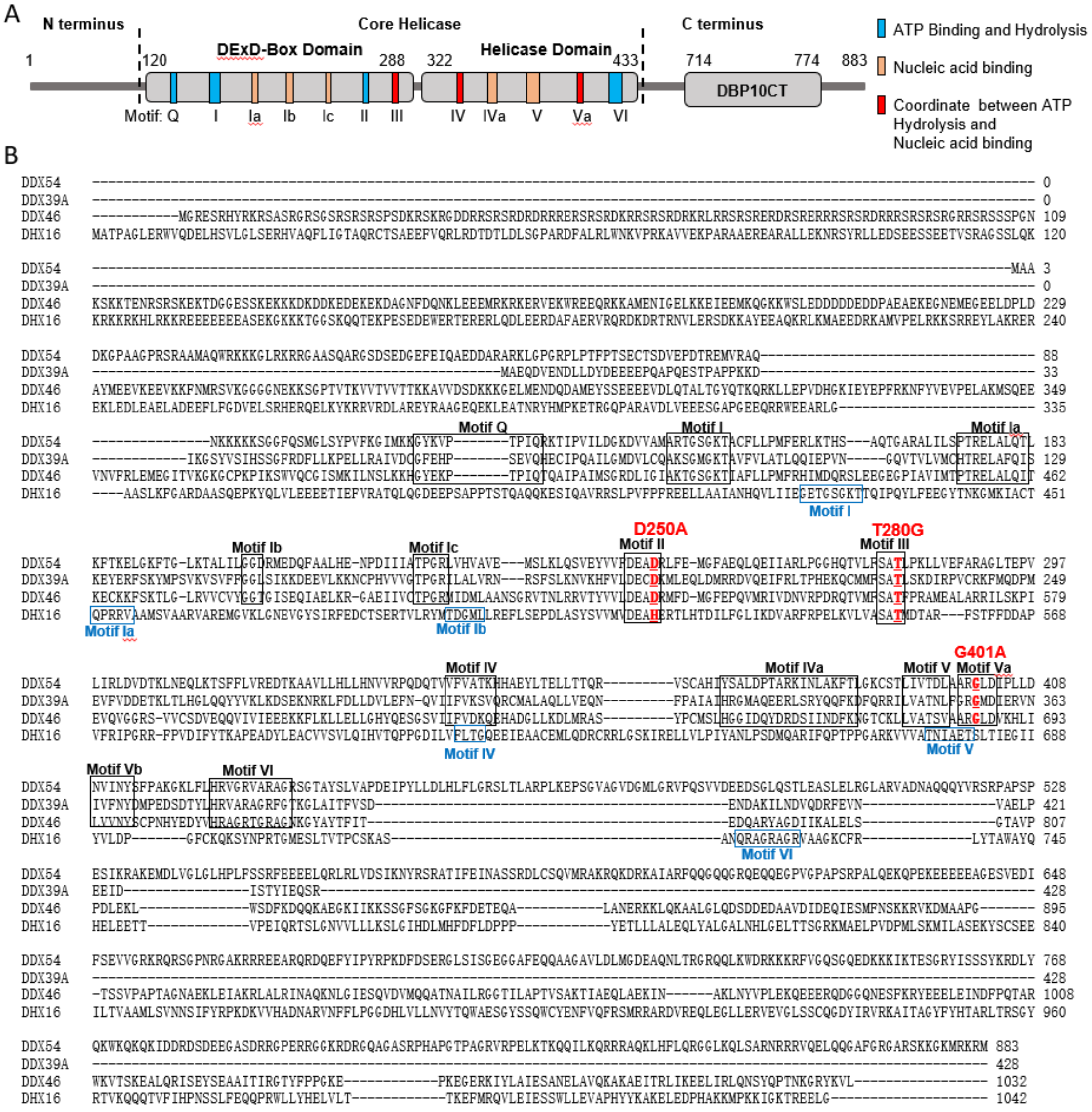

Supplementary Fig.4. Identification of key amino acids required for ATPase and helicase of DDX54 by multiple alignments. Related to Figure 8.

(A) Schematic diagrams of DDX54 protein, highlighting the core helicase domains that are composed of eight conserved motifs in DExD box helicase family.

(B) Multiple alignments of DDX54, DDX39A, DDX46 and DHX9. The characterized motifs conserved in DExD/H box helicase family members are indicated by solid boxes according to the published data for DDX46 (Zheng et al., 2017), DHX16 (Hage et al., 2022) and DDX5 (Xu et al., 2021). DExD box helicase family conserved motifs are marked with black boxes, and DExH box

helicase family conserved motifs marked with blue boxes. The red indicates the key amino acids of DDX54 that are mutated in (A) (D250A, T280G and G401A).

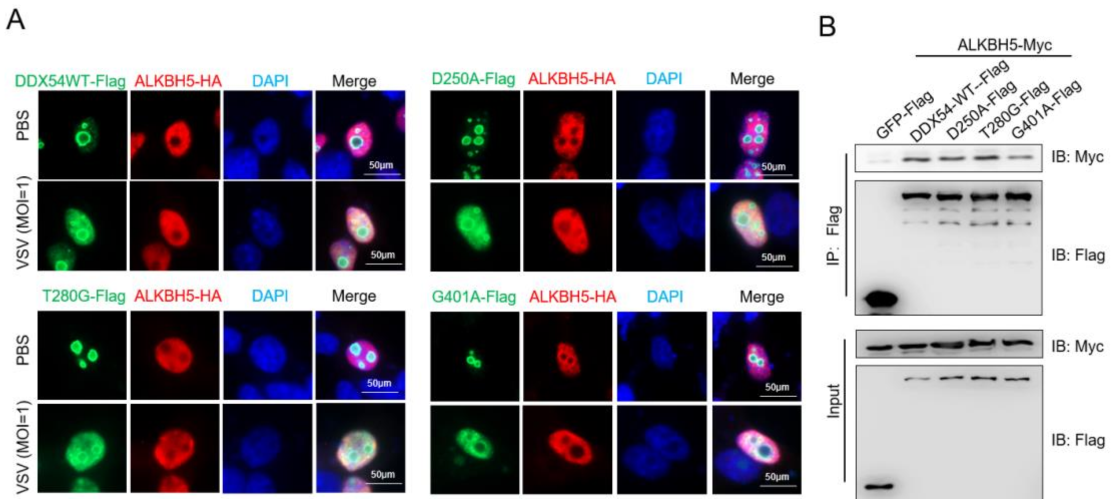

Supplementary Fig.5. Identification of key amino acids required for ATPase and helicase of DDX54 by multiple alignments. Related to Figure 8.

(A) Immunofluorescence observation of ALKBH5 binding to DDX54 or mutants in nucleoplasm upon VSV infection. HEK293T cells seeded in 6-well plate with cover slip overnight were transfected for 24h with ALKBH5-HA, together with DDX54-Flag or each of Flag-tagged mutant (1 μg each), and subsequently infected with VSV (MOI=1) for 6h, followed by immunofluorescence staining with anti-HA Ab or anti-Flag Ab.

(B) Co-IP analyses of ALKBH5 binding to DDX54 or mutants. HEK293T cells seeded in 10 cm^2 plates overnight were transfected with the indicated plasmids (5μg each). 24h later, cells were harvested for Co-IP assays with corresponding tag antibodies.

Supplementary Table 1. Primers used in this study

Primers	Sequence
vsv-F	ACGGCGTACTTCCAGATGG
vsv-R	CTCGGTTCAAGATCCAGGT
ifnb-F	TTGTTGAGAACCTCCTGGCT
ifnb-R	TGACTATGGTCCAGGCACAG
isg15-F	GAGAGGCAGCGAACTCATCTT
isg15-R	CCAGCATCTTCACCGTCAGG
ccl5-F	GCCTCCCCATATTCCTCGGA
ccl5-R	TGACCTGTGGACGACTGCTG
gapdh-F	ATTCCATGGCACCGTCAAGG
gapdh-R	ATGGTTCACACCCATGACGA
u6-F	GTGCTCGCTTCGGCAGCA
u6-R	AATATGGAACGCTTCACGAAT
ddx54-F	TCTGGAGGCTTCCAGTCCATG
ddx54-R	TCTTGAGCCGCTCGAACATTG
alkbh5-F	AGCTGGTGATCCAAAAGCTG
alkbh5-R	ACTGGTTCCGACACCCGAATA
rig-i-F	TAGGGAGGAAGAGGTGCAGT
rig-i-R	ATCGGTTGGGATAATTCTGG
mavs-F	AAGAGACCAGGGACCTCGGA
mavs-R	ACAGGCATGGGGTAACTTGG
t*bk1*-F	CGGAGACCCGGCTGGTATAA
tbk1-R	ATCCACTGGACGAAGGAAGC
irf3-F	ATCGTAGGCCGGACCATGGGAA
irf3-R	GAGGAGCGAGGGCTCAGCTCTC
irf7-F	ACACTGGTTCAACACCTGTGAC
irf7-R	TGGAGTTCTCATTAGACTGGGT
traf3-F	ACCGCGAGAACTCCTCTTTCCT
traf3-R	GGGATCGGGCAGATCCGAAG
traf6-F	ATCCAGAGTTTGCCGTCCAAGC
traf6-R	TTGAGCAAGTGAGGGCAAGCTA

References

1. Hage A, Bharaj P, van Tol S, et al. The RNA helicase DHX16 recognizes specific viral RNA to trigger RIG-I-dependent innate antiviral immunity. Cell Rep. 2022;38(10):110434. doi:10.1016/j.celrep.2022.110434.
2. Xu J, Cai Y, Ma Z, et al. The RNA helicase DDX5 promotes viral infection via regulating N6-methyladenosine levels on the DHX58 and NFκB transcripts to dampen antiviral innate immunity. PLoS Pathog. 2021;17(4):e1009530. Published 2021 Apr 28. doi:10.1371/journal.ppat.1009530.
3. Zheng Q, Hou J, Zhou Y, Li Z, Cao X. The RNA helicase DDX46 inhibits innate immunity by entrapping m6A-demethylated antiviral transcripts in the nucleus. Nat Immunol. 2017;18(10):1094-1103. doi:10.1038/ni.3830.
